# Drivers and extent of surface water occurrence in the Selenga River Delta, Russia

**DOI:** 10.1016/j.ejrh.2021.100945

**Published:** 2021-12

**Authors:** Saeid Aminjafari, Ian Brown, Sergey Chalov, Marc Simard, Charles R. Lane, Jerker Jarsjö, Mehdi Darvishi, Fernando Jaramillo

**Affiliations:** a Department of Physical Geography and Bolin Centre for Climate Research, Stockholm University, Stockholm SE-106 91, Sweden; b Faculty of Geography, Lomonosov Moscow State University, Moscow 119991, Russia; c Jet Propulsion Laboratory, California Institute of Technology, Pasadena, CA 91109, USA; d Office of Research and Development, US Environmental Protection Agency, Athens, GA 45268, USA; e Baltic Sea Centre and Stockholm Resilience Center, Stockholm University, Stockholm SE-106 91, Sweden

**Keywords:** Surface water occurrence, Selenga River Delta, Supervised classification

## Abstract

**Study region:**

Selenga River Delta (SRD), Russia.

**Study focus:**

How is water occurrence changing in the SRD, and what are the hydroclimatic drivers behind these changes? The presence of water on the surface in river deltas is governed by land use, geomorphology, and the flux of water to and from the Delta. We trained an accurate image classification of the Landsat satellite imagery during the last 33 years to quantify surface water occurrence and its changes in the SRD. After comparing our estimations with global-scale datasets, we determined the hydrological drivers of these changes.

**New hydrological insights for the region:**

We find mild decreases in water occurrence in 51% of the SRD’s surface area from 1987–2002 to 2003–2020. Water occurrence in the most affected areas decreased by 20% and in the most water-gaining areas increased by 10%. We find a significant relationship between water occurrence and runoff (R^2^ = 0.56) that does not exist between water occurrence and suspended sediment concentration (SSC), Lake Baikal’s water level, and potential evapotranspiration. The time series of water occurrence follows the peaks in the runoff but not its long-term trend. However, the extremes in SSC do not influence surface water occurrence (R^2^ < 0.1), although their long-term trends are similar. Contrary to expected, we find that the Delta has a relatively stable long-term water availability for the time being.

## 1. Introduction

River deltas provide ecosystem services to humans such as freshwater storage, pollutant retention and attenuation, recreational activities, flood control, and fishing (Day et al., 2019). They also help to achieve sustainable development (Jaramillo et al., 2019). Despite their importance in providing these services, they are under pressure from human activities and the effects of human-induced climate change (Day et al., 2019; Lin et al., 2017; Wu et al., 2020; Zhou et al., 2018). Upstream water impoundment and regulation decrease sediment discharge and flatten runoff peaks, necessary for hydraulic flushing and wetland sheet flow (Syvitski et al., 2009; Wu et al., 2020). Water extraction, excessive groundwater use, and methane extraction have led to decreased incoming water flows, sediment compaction, and subsidence in deltas throughout the world (Giosan et al., 2014). Giesen (2020) and Nienhuis et al. (2020) emphasize that local focus on deltas with a large set of ecosystem services is necessary to understand and manage deltas’ hydrological and morphological changes.

Change in surface water occurrence can be the response of anthropogenic and climatic drivers in deltas. Surface water occurrence is defined as the presence of water at a specific location on the surface and at a particular time. For the particular case of deltas, water occurrence can be permanent in open water areas such as main river channels, streams, and in-stream wetlands, or temporary such as sandbanks, flooded wetlands, and floodplains. Apart from the direct effects of human activities, water occurrence changes are also driven by in-stream hydraulics, fluvial geomorphologic processes, and sediment transport. Therefore, understanding changes in hydrological connectivity and patchiness resulting from the spatial and temporal distribution of surface water occurrence is relevant for the conservation of deltaic ecosystems (Cui et al., 2020).

The change in surface water occurrence is spatially heterogeneous, and its direction of change, loss or gain, is case-specific (Borja et al., 2020). While global-scale studies are very relevant for assessing changes in surface water resources at large scales (e.g. Donchyts et al., 2016; Pekel et al., 2016), they may miss a detailed understanding of local and case-specific changes in water occurrence. Furthermore, most studies of changes in water occurrence do not focus on identifying the most relevant hydrological drivers of change. For instance, water occurrence changes in a delta may be related to the main river’s incoming water and sediment discharge, evapotranspiration from the surface water, or changing tides.

We here perform an analysis of change in water occurrence between 1987–2002 and 2003–2020 in the Selenga River Delta (SRD), a lacustrine delta covering 540 km^2^. The Delta plays a vital hydrological role for Lake Baikal. For example, the SRD acts as an active sink of various metals, reducing 77–99% concentrations during moderate and high flow conditions (Chalov et al., 2017b). Shinkareva et al. (2019) also showed that seasonally flooded regions control the total metal concentrations flowing into Lake Baikal. Hence, assessing changes in water occurrence in the Delta serves as a diagnostic of these hydrological functions. Severe droughts during the last two decades in Mongolia and Transbaikalia have decreased the discharge of the Selenga River (Antokhina et al., 2019; Frolova et al., 2017), with potential changes in water occurrence within the Delta. Our primary research question is: How is water occurrence changing in the Selenga River Delta, and what are the hydroclimatic drivers behind these changes? We aim to determine the magnitude of change in water occurrence in the Delta over more than 30 years and relate these changes with the hydroclimatic fluxes and processes that regulate water availability in the Delta, including the runoff and sediment concentration in Selenga River hydrological basin, evapotranspiration, and water level changes in the Lake Baikal.

Satellite observations are valuable sources to understand changes in water occurrence (Allen and Pavelsky, 2018; Borja et al., 2020; Chini et al., 2017; Donchyts et al., 2016; Guo et al., 2017; Pekel et al., 2016; Zhang et al., 2017). With more than 35 years of medium-resolution acquisitions, the Landsat project is a convenient source of optical imagery to monitor water occurrence changes, with even several images available per month for specific deltas. In addition, advanced computational algorithms and cloud-based platforms enable processing large amounts of data in relatively short periods, providing a high-spatial resolution of changes. To answer the research question, we specifically train the Landsat data to detect a more case-specific representation of water occurrence within the Delta, generate a time series of change in water occurrence, and compare our results against already available global datasets of water occurrence that do not train the data. The two hypotheses driving the study are:

1. Changes in water occurrence in the Selenga River Delta follow runoff oscillations occurring during the last 30 years, as in other Deltas worldwide.
2. A direct relationship between water occurrence in the Delta and runoff and suspended sediment concentration is more evident than with evapotranspiration and Lake Baikal water level, as the lake water level is regulated by Irkutsk dam on the main outflow of Lake Baikal-Angara River since 1959, and evapotranspiration from the Delta is low.

## 2. Materials and methods

### 2.1. Study area

**Fig. 1.**
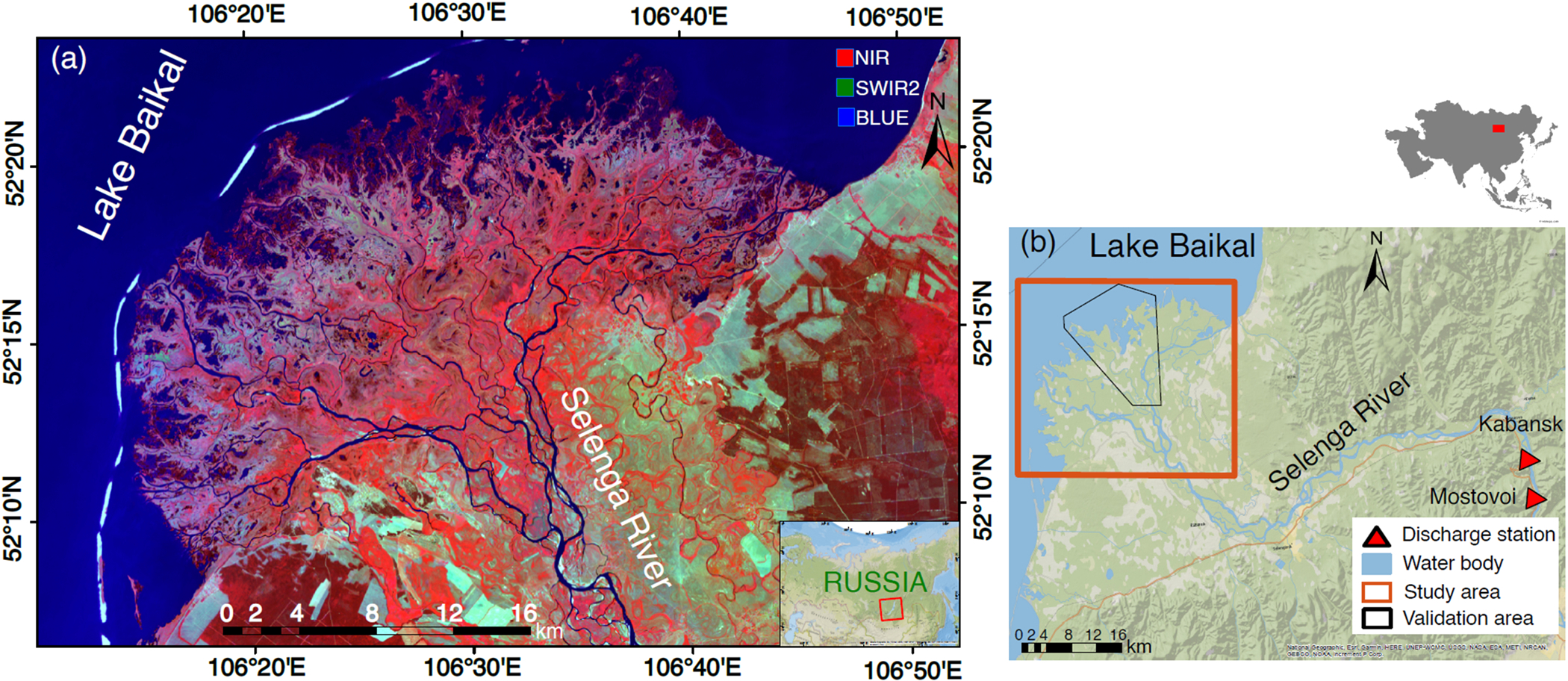
The Selenga River Delta. (a) The Selenga River Delta location in Russia over a false-color Landsat 8 image acquired on 23 June 2017, path 132, frame 24. Water (blue), vegetation is shown by values in Near-Infrared (NIR) and urban areas are in Short-Wave Infrared (SWIR2) range; (b) Discharge measuring stations (red triangles) and extent of the high-resolution land cover classification (solid black-line border) available by Berhane et al. (2018) used here to validate the results of water occurrence. (For interpretation of the references to color in this figure legend, the reader is referred to the web version of this article.)

**Fig. 2.**
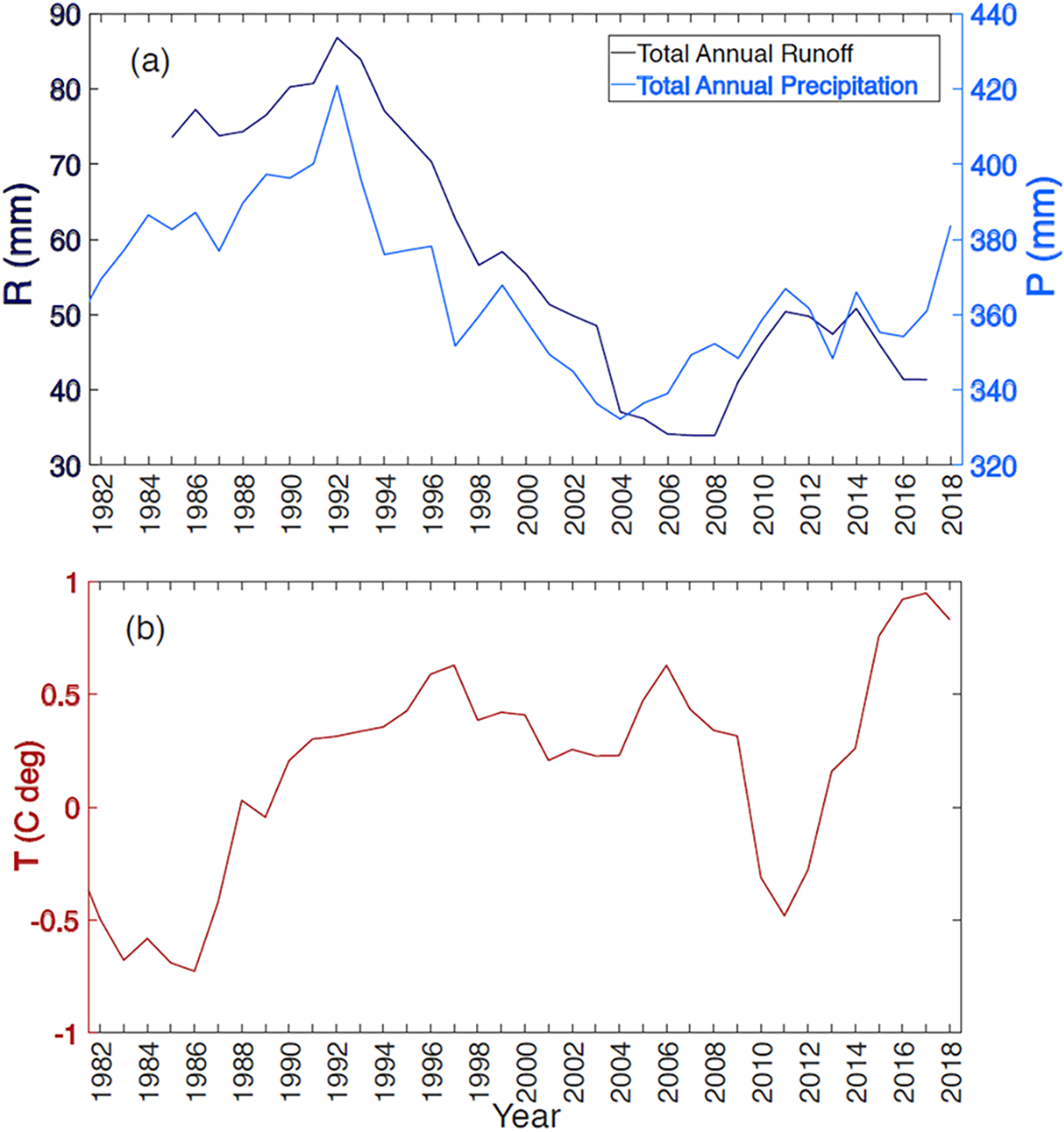
Five-year moving averages of (a) monthly runoff (R), precipitation (P), and (b) temperature (T) in the SRD (For the runoff calculations, we divided discharge data at station Mostovoi by the upstream hydrological basin of 440,200 km^2^, of which 67% falls in Mongolia and 33% in Russia.) See Section 2.5 for sources.

The Selenga River Delta is located in eastern Russia (Fig. 1) along Lake Baikal’s southern shore. Out of around 365 other rivers, the Selenga River is the main river flowing into Lake Baikal. It is responsible for almost 50% of the runoff water and 60% of the transported sediments into the lake system, and its hydrological basin covers more than 82% of the Lake’s basin (Potemkina., 2004). The SRD’s unique habitats and ecosystem services have made this Delta a Ramsar Wetland of International Importance (Berhane et al., 2018; Lane et al., 2015). The Delta covers 540 km^2^ and receives 315 mm of annual precipitation, concentrated from April to October, causing floods and freshets in the main channel and tributaries. The continental climate is described by temperature variations between + 14 °C on average in July and − 19 °C in January, with a growing season of around 150 days starts in mid-May (Fig. S1) (Berhane et al., 2018; Lane et al., 2015). Precipitation and runoff in the Selenga River Basin have decreased from the highest reported peak in 1992 to its lowest between 2004 and 2008 (Fig. 2a). Air temperature in the SRD increased to the highest mean annual values in 2016 and 2017 (Fig. 2b).

Being a lacustrine river delta, changes in water occurrence in this Delta are not related to rising sea levels. Instead, changes in the Selenga River discharge and Lake Baikal water level are related to hydroclimatic data (Antokhina et al., 2019) and fluvial geomorphological processes (Pietroń et al., 2018; Dong et al., 2019; Shinkareva et al., 2019). The Selenga River faces several socioeconomic and environmental impacts, influencing Lake Baikal (Borisova, 2019). The anthropogenic impact on ecosystems in the Selenga River basin has increased in the recent decades, e.g., by the extraction of minerals, primarily gold, urbanization, and agricultural development, especially in the upper Mongolian part of the basin (Jarsjö et al., 2017; Garmaev et al., 2019). The long-term low water period observed in the region (Gelfan and Millionshchikova, 2018) has a significant impact on delta processes and wetland-dominated areas of the SRD, also posing drastic changes in sediment transport and water quality (Chalov et al., 2017b; Shinkareva et al., 2019).

### 2.2. Satellite data and classification

Unsupervised classification methodologies can determine long-term global water occurrence changes, mostly without training data (Borja et al., 2020; Donchyts et al., 2016; Pekel et al., 2016). However, for local studies, training data or pre-existing knowledge in the spatial distribution of water occurrence can improve prediction and avoid misclassifications. Misclassifications occur when land cover classes do not correspond to the spectral clusters from unsupervised classification (Lane et al., 2015). For example, in deltas with complex structures and diverse vegetation types, surface water is likely misclassified as surrounding vegetation or vice versa. To develop a time series of water occurrence, training large datasets of historical satellite images is unfeasible and undesirable due to the forced coarseness of the data granularity. However, for the case studies of single deltas, time series analysis has fewer requirements and more fine-grained spatial and temporal resolutions, providing information on the drivers and extent of changes in water occurrence in the deltas.

We mapped the surface water occurrence in the SRD in a time series of 30-m pixel size Landsat imagery from 1987 to 2020. The entire spatial extent of images covers ~1256 km^2^ of the area (the Delta and a section of the Lake). The classification data analyses were done using ENVI version 5.5 (Exelis Visual Information Solutions, Boulder, Colorado). We used Landsat Level-2 Surface Reflectance data from the United States Geological Survey, which provides atmospherically corrected scenes of Landsat 4–5/TM, 7/ETM+, and 8/OLI upon request (Masek et al., 2006; Vermote et al., 2016). In total, we obtained 195 images between 1987 and 2020 with less than 10% cloud cover over the scenes of the SRD but discarded more than half of the images due to Scan Line Corrector (SLC) errors (stripes on the Landsat-7 images due to instrument deficiency), cloud contamination, and geometrical errors. Other images in winter were also discarded since frozen water generates inaccurate classifications of water surfaces. For the 87 remaining images, the Normalized Difference Vegetation Index (NDVI) and Normalized Difference Water Index (NDWI) were calculated by applying Eq. (1) and Eq. (2) (McFeeters, 1996), and stacking with the Near Infrared (NIR), Shortwave Infrared (SWIR2) and the Blue band for each scene.


(1) $$NDVI=\frac{NIR\;-\;Red}{NIR\;+\;Red}$$

(2) $$NDWI=\frac{\;Green\;-\;NIR}{\;Green\;+\;NIR}$$

The Red and Green bands represent reflectance in the visible red and the spectrum’s visible green bands. Since the highest reflectance difference between water and vegetation occurs in these bands and indices, using their combination in the classifier makes it possible to distinguish open water from the surrounding vegetation and land. For example, water bodies have 0.5 < NDWI < 1 and NDVI ≈ − 1, whereas vegetation has 0 < NDWI < 0.2 and 0.2 < NDVI < 1. Zhou et al. (2017) assessed different indices’ performance for differentiation of water surfaces and concluded that the NDWI-based algorithms (such as the one we used here) outperform other algorithms. Therefore, the information in the mentioned band-index combinations is considered to fulfill our goal of mapping the Delta.

We used the supervised Maximum Likelihood classification approach to assess the habitats of the SRD. Before the supervised Maximum Likelihood classifier was applied on the stacked bands, we selected the training data of water surface recognition in each image by the visual inspection of the Google Earth’s historical view on the corresponding date and the true-color composites of each scene (visible Red, Green, and Blue bands).

The binary classification for all available images results in class images containing two classes of pixels: water (equals 1) and non-water (equals 0). The training dataset used for each classification process has 245,000 pixels (~220 km^2^), which corresponds to 18% of the whole image extent. Therefore, we prepared two sets of the land-water dataset, one for training the classifier and the other for calculating the confusion Matrix and the classification accuracy. We chose the land-water data sets in similar areas for all images to avoid misinterpretation as long as the delineation borders fitted the water bodies’ exact true-color locations. As a result, the overall accuracy of classification is higher than 98% (Table S1). The Maximum Likelihood supervised classifier is widely applied to satellite imagery for land cover mapping, and it provides satisfactory classification accuracy and processing speeds. (e.g. Bao and Ren, 2011; Guo et al., 2017). This algorithm assumes that the pixel classes usually are distributed in the spectral space based on the training data. It calculates the probability of a pixel belonging to a specific landcover class (*c*_*m*_) when the probability (*p*) of a pixel with a given value (*d*) of belonging to *c*_*m*_ is higher than of belonging to another class *c*_*n*_ (Richards, 1999; Eq. (3)).


(3) $$d\in c_{m}\;if\;\;p\left(c_{m}|d\right)>p\left(c_{n}|d\right)\;\;for\;\;\;all\;\;n\neq m$$

### 2.3. Surface water occurrence and its changes

We classified each pixel binarily for each image and date based on the supervised Maximum Likelihood classifier results, either 0 (water) or 1 (not water). Since the non-vegetation dry lands might be misclassified with the pixels identified as water, we included the sand bars and other non-vegetation dry areas in the training data to avoid this problem.

We introduce five parameters of water occurrence and explain their calculation in this section: (1) the spatial mean of water occurrence over each image scene $\left({\bar{w}}_{s}\right)$, (2) the monthly mean of water occurrence at each pixel (*w*_*m*_), (3) the mean water occurrence at each pixel for the whole period $\left(\bar{w}\right)$, (4) the monthly change in water occurrence at each pixel between the two periods 1987–2002 and 2003–2020 $\left(\bar{\Delta w}\right)$, and (5) the mean magnitude of change in water occurrence at each pixel between the two periods $\left(\bar{\Delta w}\right)$. For the change in water occurrence during the last 33 years, we choose equally long periods (16.5 years) to portray the long-term hydroclimatic changes within the study period, and even so, this study does not aim to capture interannual changes from year to year. As an example, Zamora et al. (2020) and Piemontese et al. (2019) showed that equal long-term periods could be used to understand long-term hydroclimatic changes in water resources such as the Selenga River and its Delta.

To compare the water occurrence time series with the potential hydrological drivers of change, we needed a single value of water occurrence for each image. To get that value, we spatially averaged the water occurrence of all pixels of each image to obtain the mean water occurrence (${\bar{w}}_{s}$: subscript s represents the spatial mean) and multiplied by 100 to get the values in percentage:

(4) $${\bar{w}}_{s}=\frac{\sum_{i=1}^{r}v_{i,j}}{r}*100(\%)$$

where *v*_*i*_,_*j*_ is the water occurrence of the *i*^*th*^ pixel in the class image *j* with the total number of pixels *r*.

We followed Pekel et al. (2016) to estimate water occurrence on a given land surface in the SRD during the 33 years of 1987–2019. We calculated each pixel’s mean of the binary values of all images for that specific pixel, leading to a final value between zero and one. Since not all months had the same number of images available, our interpretation of water occurrence is more biased towards the summer and autumn periods that contain more images due to their favorable meteorological conditions. For an unbiased analysis, we calculated a mean value of water occurrence for each month in each pixel during the same period (*w*_*m*_)

(5) $$w_{m}=\frac{\sum_{j=1}^{n}v_{i,j}}{n}$$

where *m* is the month, *j* is a class image from the total class images available for that specific month (*n*) from January 1987 to December 2019. While images taken in the summer and autumn periods have a higher quality and lower presence of clouds, images taken during December, January, February, and March were discarded as Lake Baikal, and some parts of Delta are covered by ice and snow.

The mean water occurrence at each pixel and for the whole period $\left(\bar{w}\right)$ is calculated by averaging the water occurrence of 8 months (*w*_*m*_) and multiplying by 100 to have the values in percentage as:

(6) $$\bar{w}=\frac{\sum_{m=1}^{n=8}w_{m}}{n}*100(\%)$$

To understand the temporal change in water occurrence for each month of the year (*Δw*_*m*_), we subtracted the mean *w*_*m*_ of the period 1987–2002 (*w*_*m*1_) from that of the period 2003–2020 (*w*_*m*2_) as:

(7) $$\Delta w_{m}=w_{m2}-w_{m1}$$

Finally, the mean magnitude of change in water occurrence $\left(\bar{\Delta w}\right)$ per pixel was calculated by averaging the monthly changes of water occurrence *Δw*_*m*_ as:

(8) $$\bar{\Delta w}=\frac{\sum_{m=1}^{n=8}\Delta w_{m}}{n}$$

As *w*_*m*_ ranges from zero to one, *Δw*_*m*_ and $\bar{\Delta w}$ do from − 1 to + 1, where a value of − 1 means no water in any class image in the second period and water in all class images in the first period, and vice versa. Therefore, $\bar{\Delta w}$ provides information about the expansion and shrinkage of the water surface area, and negative and positive values correspond to decreasing and increasing water occurrence, respectively.

### 2.4. Validation and comparison with previous studies

Berhane et al. (2018) performed three supervised classification methods (decision-tree, rule-based, and random forest) in the SRD based on high-resolution multispectral imagery of the WorldView-2 sensor with 2-meter pixel size in June 2011. They trained and validated their results with 228 field data points to obtain a class image of 22 wetland classes (Fig. 1b and S2). We extracted the water bodies without vegetation or very sparse floating vascular vegetation and made a binary class image of water/non-water classes. We compared this image with the corresponding class image in the same period (June 2011) and calculated six accuracy assessment parameters in an area of approximately 140 km^2^ for 10,000 equally distributed random points (i.e. User’s Accuracy (UA), Producer’s Accuracy (PA), Overall Accuracy (OA), F-score, Kappa, and Matthews Correlation Coefficient (MCC); Table S2).

To emphasize the importance of the training data in binary classification in wetlands with different vegetation types, we compare the accuracy of our study with the global study by Pekel et al. (2016). Pekel et al. (2016) applied unsupervised Expert systems classifiers on historical Landsat images on a global scale to obtain water occurrence and its changes during the lifetime of Landsat imagery worldwide. We downloaded the mean class image of their product in June 2011 via the Google Earth Engine platform and calculated the accuracy assessment measures of their results with respect to the reference class image of Berhane et al. (2018).

Finally, we compare the change from permanent land to permanent water and vice versa obtained in this study with Pekel et al. (2016) and Donchyts et al. (2016). The latter is also based on Landsat images’ long-term availability and performs an unsupervised classification based on thresholding NDWI and NDVI, with the final product of change from land to water accessible through Google Earth Engine on the users’ preferences.

### 2.5. Hydroclimatic data and landcover map

We analyzed four hydrological variables: The Selenga River’s surface runoff, the water level in Lake Baikal, suspended sediment concentration (SSC; in the absence of bedload discharge data), and potential evapotranspiration. We used daily river discharge (1987–2017) and 10-day SSC (1990–2017) from the Russian Federal Service for Hydrometeorology and Environmental Monitoring (Roshydromet) in the gauging stations closest to the Delta (Mostovoi: discharge and SSC, and Kabansk: discharge). Mostovoi and Kabansk stations are located ~127 km and ~43 km upstream of the Delta. We also extracted temperature, precipitation, and potential evapotranspiration data for the region of the SRD, between 1982 and 2020, from the 0.5° by 0.5° gridded data sets of the CRU of the Climatic Research Unit (Harris et al., 2020). For the case of precipitation, we used the mean monthly precipitation values of all cells falling in the Selenga River hydrological basin upstream of each of the two discharge stations (basin area = 445,000 km^2^). To make surface water occurrence and runoff data comparable, we calculated the 10- and 5-day averages of runoff data before, after, and on the images’ acquisition date and time.

For Lake Baikal’s water level, we used two gauge stations of the International Data Centre on Hydrology of Lakes and Reservoirs (Hydrolare) from 1963 to 2015, one roughly 60 km southwest of the Delta (coastal Babushkin station) and another one ~200 km northeast of the Delta (Ushkanij station on the Ushkan Islands, the archipelago on Lake Baikal), together with the processed satellite altimetric data of water level from the Hydroweb service available since 1992 and continuously updated (Crétaux et al., 2011). The altimeters used in this dataset are Topex-Poseidon, Jason, Jason-2, Jason-3, and Sentinel-3A, and the point of observation is in the middle of the lake and near the Ushkanij gauge station, which is roughly 200 km away from the Delta. Before 2014, the temporal resolution of this dataset was over 10 days, and since 2014 new altimetric satellites provide 1-day resolution water level data.

**Fig. 3.**
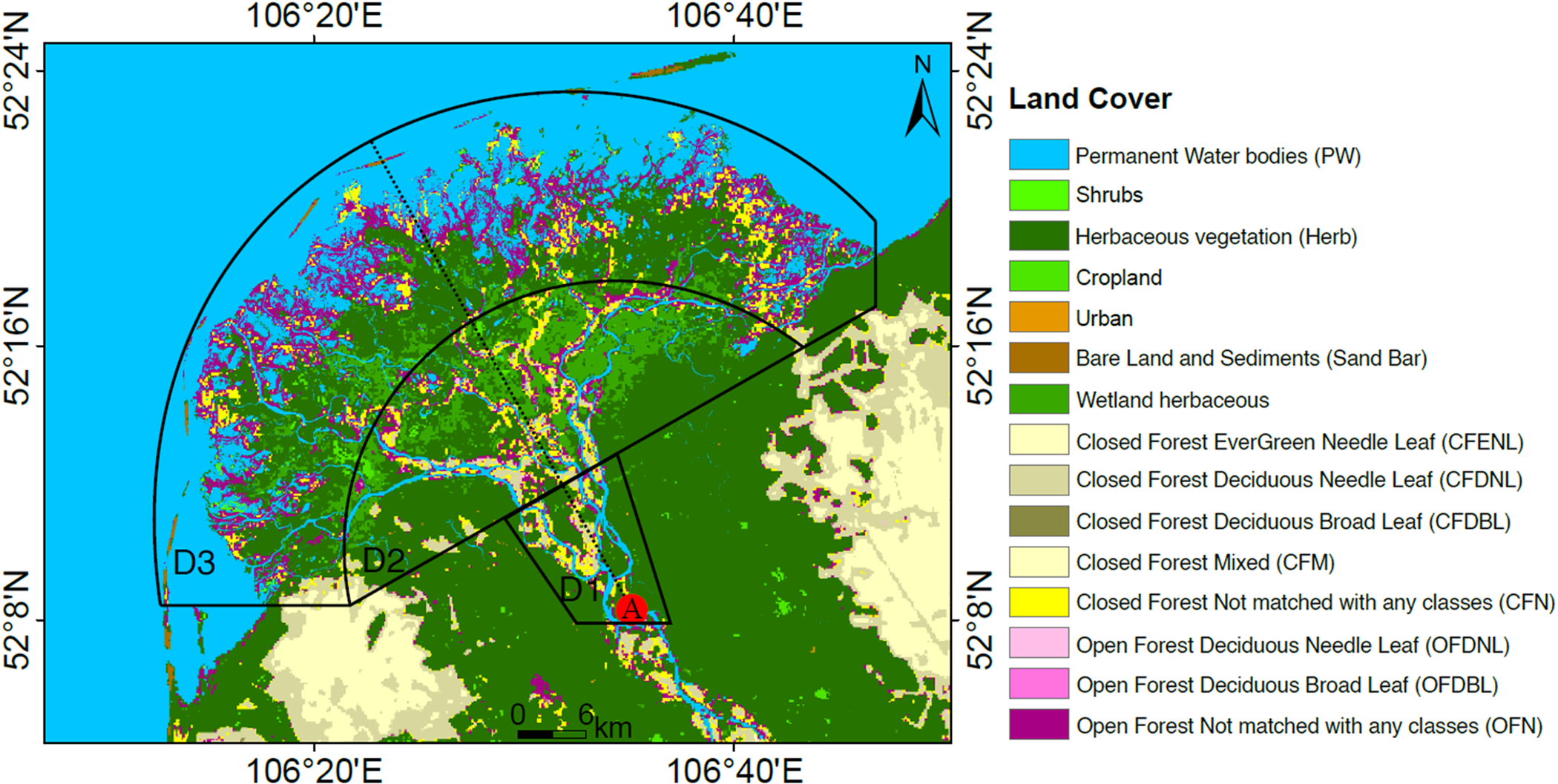
Land cover (CGLS-LC100) and three 10-km-wide (at their center) focus regions in the Delta (D1, D2, and D3). Point A is near the bifurcation of the river that we consider the head of the Delta here.

We divided the Delta into three different regions of analysis (D1: river bifurcation, D2: central portion, and D3: lake-ward, respectively; Fig. 3) to isolate the effects (on water occurrence in the Delta) of upstream runoff of the Selenga River Delta and the fluctuations of water level in Lake Baikal. We hypothesize that changes in water occurrence in the lake affect more water occurrence in the outer region D3 and the main channel of the Selenga River on the upstream region D1. To analyze the spatial variability of water occurrence, we compared water occurrence and its changes between the different land cover ecosystems in the Delta (i.e., permanent water bodies, seasonally inundated areas, wetlands, and forests; Fig. 3). We preliminarily assigned a landcover category to each pixel based on their $\bar{w}$ values; pixels with $\bar{w}>80\%$ falling into the permanent water category, pixels with $\bar{w}<10\%$ into dry lands and $10\%<\bar{w}<80\%$ as seasonal water bodies. Finally, we merged this preliminary water occurrence categorization with the dynamic land cover map of the Copernicus Global Land Service at 100-m resolution (CGLS-LC100) (Buchhorn et al., 2019) (Fig. 3). This product, obtained via Google Earth Engine, has global coverage and its reference year is 2015.

## 3. Results

### 3.1. Surface water occurrence and its changes

**Fig. 4.**
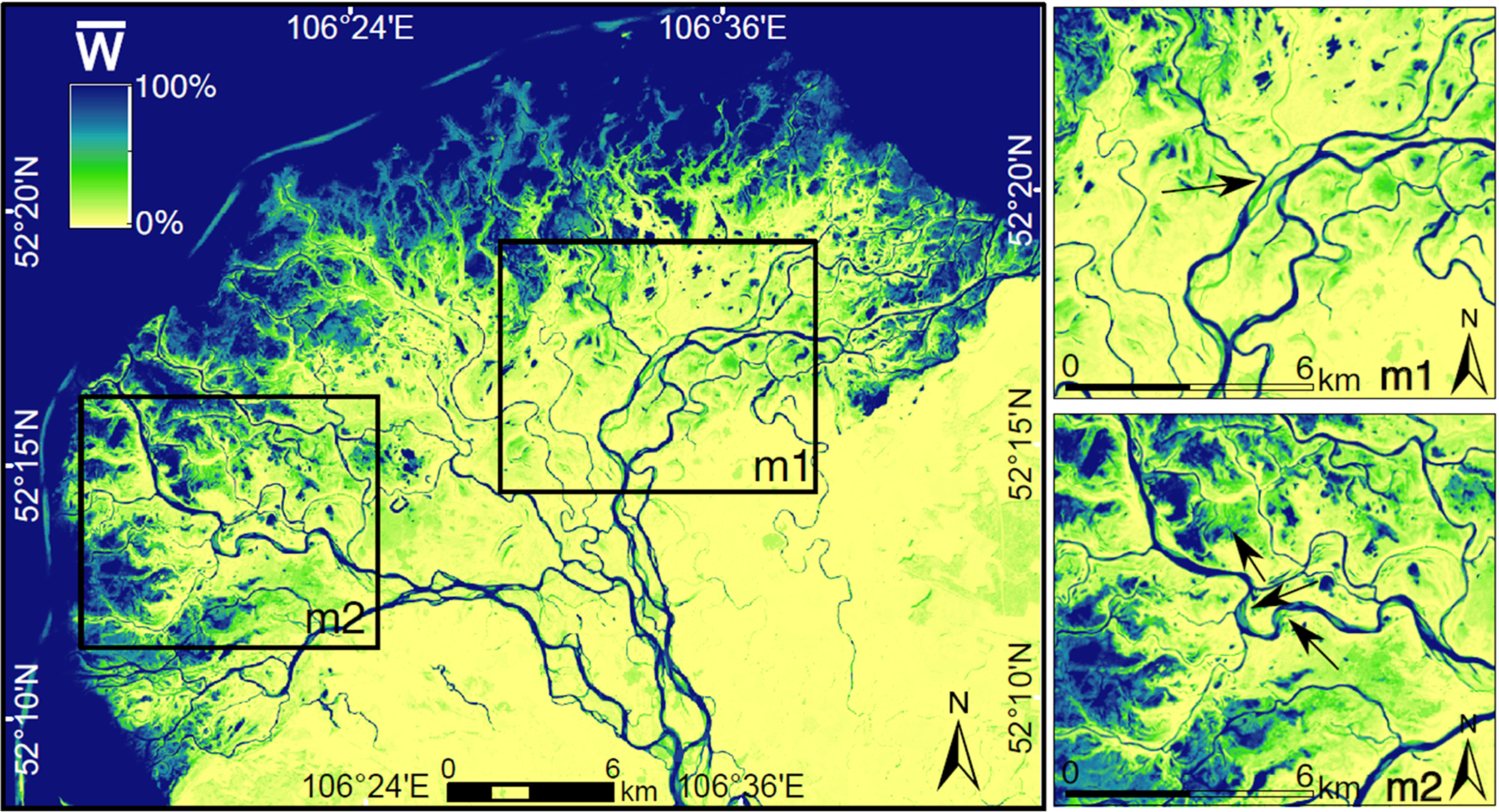
Mean surface water occurrence $\left(\bar{w}\right)$ in 1987–2020 with pixels not holding water in any class images (0%; yellow) and holding water in all class images (100%; dark blue). The arrows in zoom panel m2 show river bends and flood-prone areas with high water occurrence, yet less than permanent water bodies. (For interpretation of the references to color in this figure legend, the reader is referred to the web version of this article.)

**Fig. 5.**
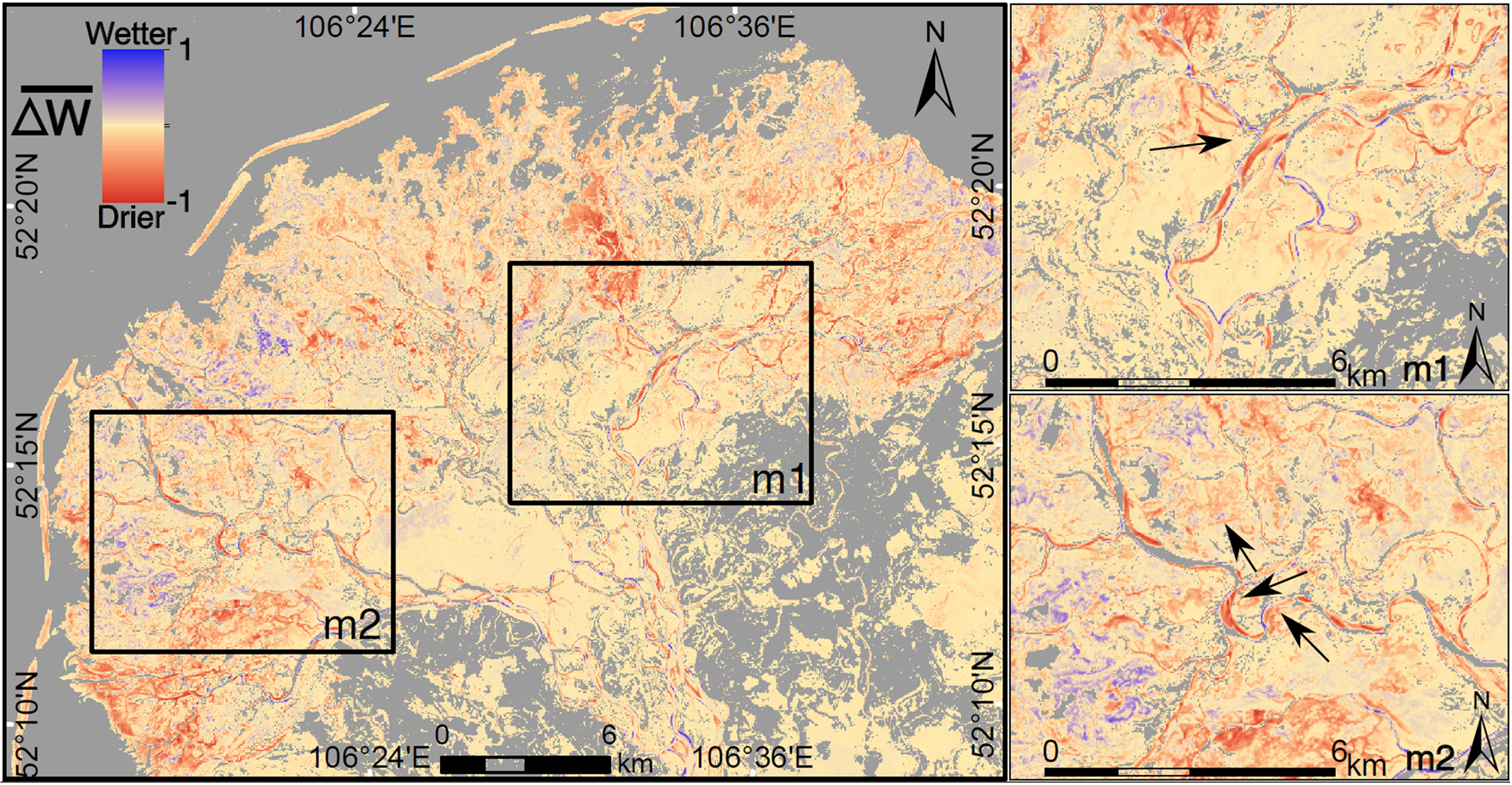
Change in surface water occurrence $\left(\bar{\Delta w}\right)$ between 1987–2002 and 2003–2020. Red areas (−1) show losses of water surface, and blue areas (+1). The arrow in zoom panel m1 show a change in flow direction from the NE (red, indicating change) to the NW (blue, indicating the increase in surface water occurrence). We masked out pixels with no change between two periods $\left(\bar{\Delta w}=0\right)$ .. (For interpretation of the references to color in this figure legend, the reader is referred to the web version of this article.)

The map of mean water occurrence $\bar{w}$ (Fig. 4) visualizes the stream network and the areas susceptible to seasonal flooding. As expected, areas along the coast of Lake Baikal have a higher water occurrence than inland. The change in water occurrence between the two periods $\bar{\Delta w}$ (Fig. 5) shows the expansion and shrinkage of the surface water areas attributed to river planform migration, newly formed or dried out streams and lakes, and the flooding of flood-prone areas. Table S3 and Fig. S3 show the number of images per month and year used to produce the water occurrence map and its changes. In general, negative values of $\bar{\Delta w}$ (orange in Fig. 5) dominate 51% of the Delta’s area and are concentrated in the southwest and eastern sections, where surface water has decreased mostly. However, the changes in water occurrence from 1987–2002 to 2003–2020 $\left(\bar{\Delta w}\right)$ are within the range − 0.2 and + 0.1, indicating a slight change in surface water occurrence.

A first look into the spatial distribution of $\bar{\Delta w}$ highlights a decreased water occurrence in the outer sediment banks in Lake Baikal, possibly due to the accumulation of sediments (Fig. 5). Decreasing $\bar{\Delta w}$ in close vicinity of the main channel and streams’ bends represents river planform migration, visualized in the zoomed panels of Figs. 4 and 5 (m1 and m2). In m1, the change $\left(\bar{\Delta w}\right)$ shows the old (north-eastern direction) and new (north-western direction) paths of a river branch due to changes in water occurrence. The availability of the images does not allow to determine the day when the change took place; however, the class images available between 1999 and 2001 show the traces of the newly-formed paths (m1). Instead, in panel m2, $\bar{w}$ is high but still less than that of permanent water bodies, signaling the presence of river bends and seasonal water bodies. Also, locations where the magnitude $\left(\bar{\Delta w}\right)$ is high in the river network (close to − 1 or +1) can represent meandering processes leading to oxbow lake formation.

**Fig. 6.**
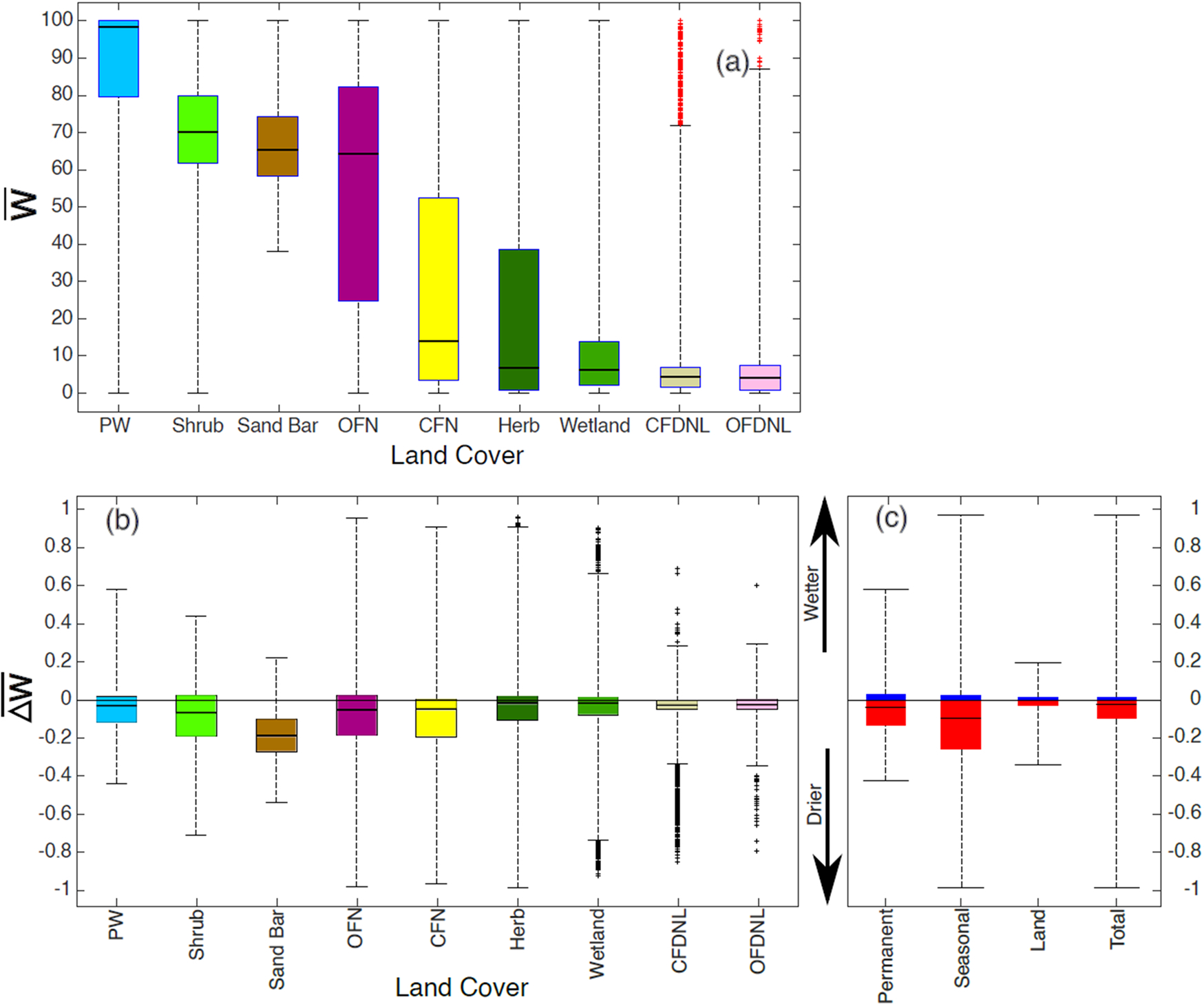
Pixel distributions of (a) mean surface water occurrence $\bar{w}$, (b) and its change $\bar{\Delta w}$ by land cover and (c) $\bar{\Delta w}$ by general categories (i.e. permanent water, seasonal water, and land). Crosses are the outliers. Abbreviations: Permanent Water bodies (PW), Bare Land and Sediments (Sand Bar), Open Forest Not matched with any classes (OFN), Closed Forest Not matched with any classes (CFN), Herbaceous vegetation (Herb), Wetland herbaceous (Wetland), Closed Forest Deciduous Needle Leaf (CFDNL), Open Forest Deciduous Needle Leaf (OFDNL).

Based on the CGLS-LC100 landcover map shown in Fig. 3, we summarized the distribution of $\bar{w}$ and $\bar{\Delta w}$ by landcover (Fig. 6a,b). The decrease in surface water occurrence is more recurrent and intense in seasonally flooded areas than in permanent water bodies (Fig. 6c, Table S4). Surface water occurrence has changed in 20% and 90% of the pixels of permanent and seasonally flooded areas, respectively, and 51% over the total extent of the SRD. In the river channel’s permanent water bodies, the increasing surface water occurrence near the river bends suggests regular geomorphic channel processes (Fig. 4; green). Although $\bar{w}$ in the outer bank of the SRD (the sand bar near the Lake) is larger than 60%, the negative values of $\bar{\Delta w}$ show how sand bars have become wider during the last three decades. According to Fig. 6b, the largest decreases in water occurrence can be seen on the sand bar near the lake $\left(-0.4<\bar{\Delta w}<-0.1\right)$.

Water occurrence in closed and open forests with needle-leaf trees (CFDNL and OFDNL) and perennial woody vegetation is less than 10% and relatively constant (Fig. 6a). However, due to the canopy coverage in these regions and Landsat products’ limitations, it is impossible to monitor the surface water below the canopy. For the case of the open and closed forest with unknown vegetation types (OFN and CFN, not matched with any other classes), water occurrence is high during the leaf-off season, and it drops in leaf-on months. The largest values of $\bar{w}$ are found in areas covered by herbaceous vegetation (Herb) and shrubs (the plants that are less than five meters tall), such as the wetland-dominated areas close to river banks as they are more susceptible to river overflow (Fig. 6b). However, farther from river branches, the wetland-dominated areas are less exposed to the river overflow, and thus lower water occurrence is observed due to dense vegetation cover (Fig. 6a). Although shrub areas see water in more than 60% of the images, there are small decreases in the majority of the pixels in these land covers (Fig. 6b). Therefore, we cannot rule out the increase in vegetation as the cause of decreasing water occurrence.

### 3.2. Accuracy assessment and comparison between datasets of water occurrence

We compared the results of water occurrence from the training data method used here with the data of Pekel et al. (2016) by six accuracy assessment measures by using the reference class image in June 2011 by Berhane et al. (2018). The confusion matrices derived from these comparisons are shown in Table 1.

Except for UA, (Table 1 and Fig. S4), the accuracy assessment indicators show higher accuracy values for this study than Pekel et al. (2016). Overall accuracy, Kappa, F-score, and MCC of this study are 0.81, 0.61, 0.79, and 0.62, larger than those of Pekel et al. (2016) (i.e., 0.73, 0.46, 0.67, and 0.50, respectively). Based on the reference image of Berhane et al. (2018), the number of misclassified pixels in our study covers 6% of the comparison area, and in Pekel et al. (2016), 11%. In the latter, many misclassified pixels occur along the stream branches and their banks, affecting the whole period’s average water occurrence. We downloaded the monthly historical data of Pekel et al. (2016) in the SRD region via Google Earth Engine (418 products between Mar 1984 and Jan 2020), and high cloud coverage and Scan Line Corrector (SLC) errors of Landsat-7 imagery affected 340 images, result in many pixels having a no-data value in large areas of the Delta (Fig. S5), possibly contributing to spatially biased water occurrence in the final averaged map in that study.

**Fig. 7.**
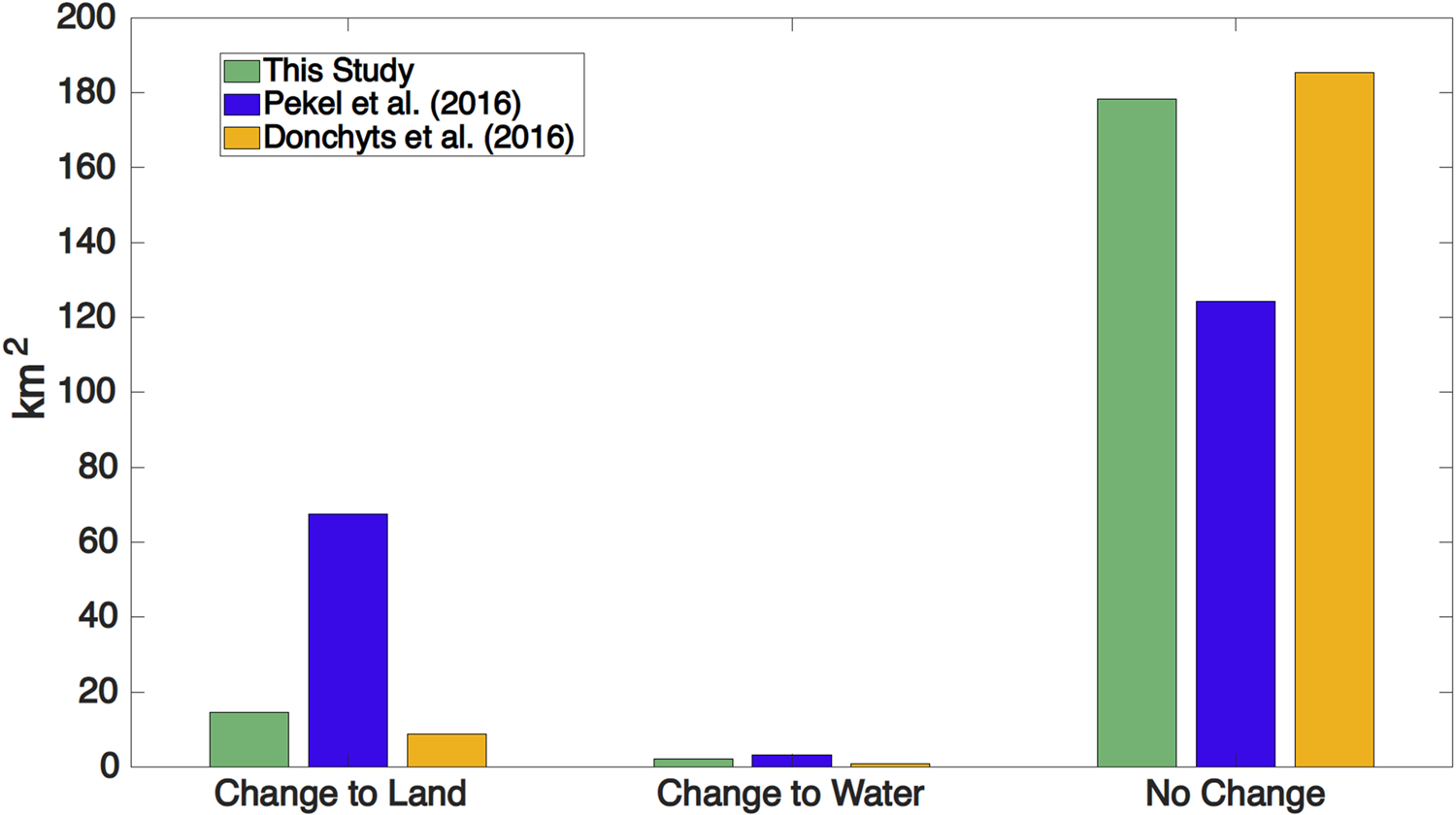
Change from water to land and vice versa for this study, Pekel et al. (2016), and Donchyts et al. (2016) in D2 region (mid-delta) shown in Fig. 3.

**Fig. 8.**
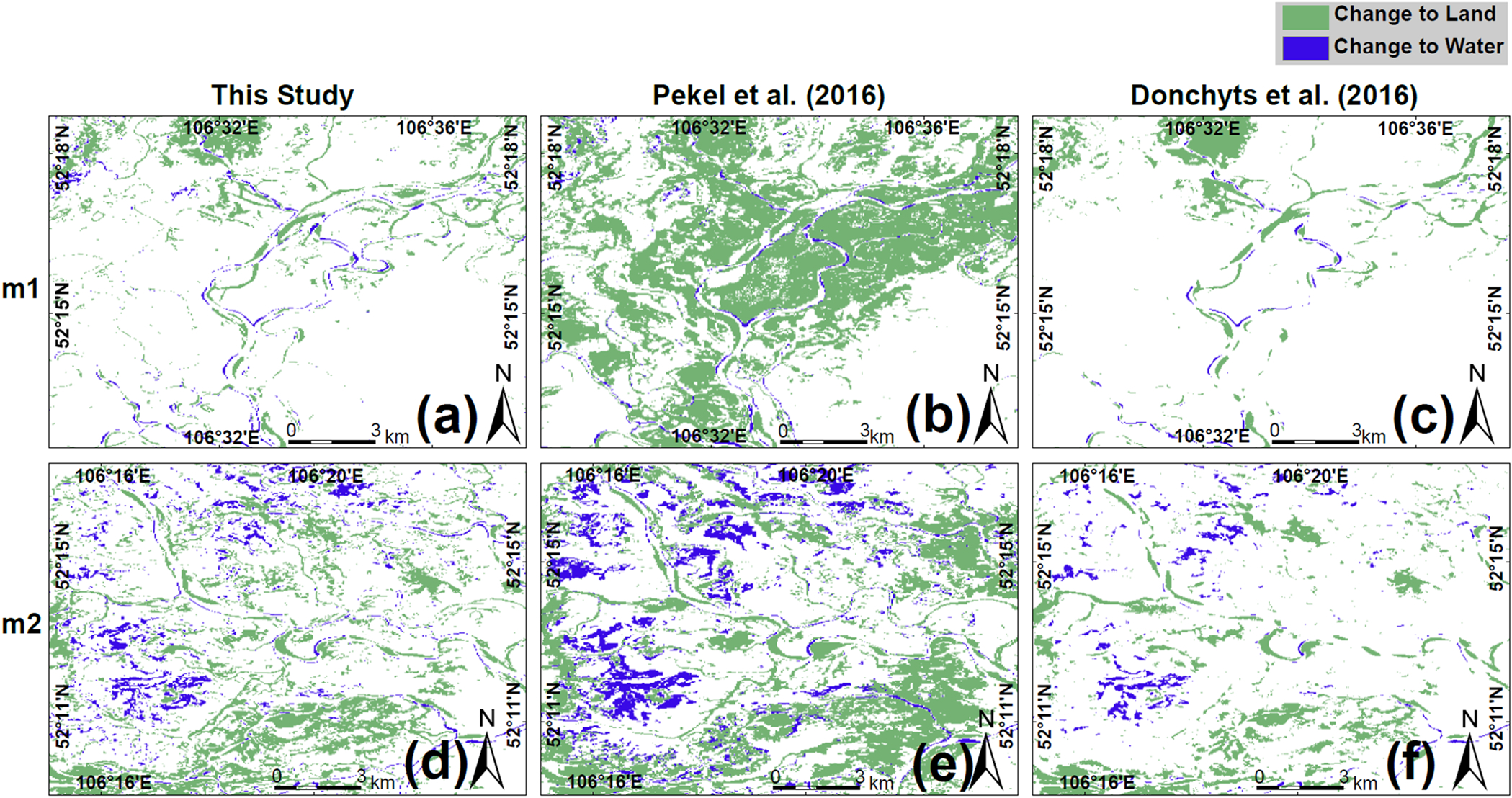
Change from water to land and vice versa in m1 and m2 regions shown on Fig. 4; (a–c) this study, Pekel et al. (2016), and Donchyts et al. (2016) respectively in m1 and; (d–f) in m2.

Now, regarding the long-term comparison of water occurrence with the Pekel et al. (2016) (between 1985–1999 and 2000–2015) and Donchyts et al. (2016) (between 1987–2002 and 2003–2020) products, Pekel et al. (2016) show more pronounced changes from water to land (Fig. 7). However, the changes from land to water along the river bends and the newly formed river branches with Donchyts et al. (2016) are less evident in both m1 and m2 than in the other two studies (Fig. 8). Therefore, the differences between our study and Donchyts et al. (2016) are negligible. Furthermore, Pekel et al. (2016) show a smaller difference between the areas where pixels change to permanent land/water and those without change (Fig. 7).

### 3.3. Relation of water occurrence to hydrological drivers

**Fig. 9.**
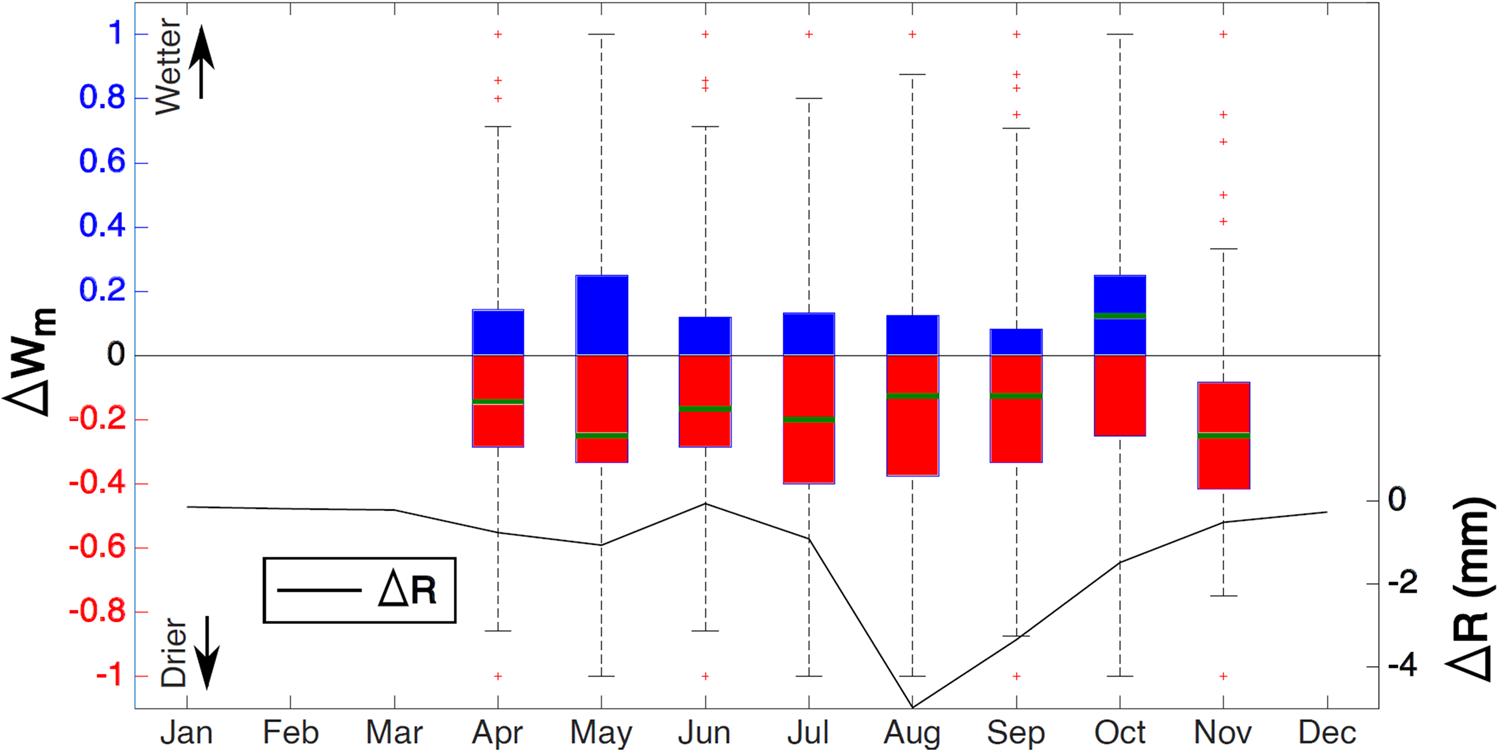
The monthly change in water occurrence $\left(\Delta w_{m}\right)$ for non-zero values and runoff in station Kabansk (Δ***R***) between the periods 1987–2002 and 2003–2020. (For interpretation of the references to color in this figure legend, the reader is referred to the web version of this article.)

The distributions of mean change in monthly water occurrence *Δw*_*m*_ between the periods 1987–2002 and 2003–2020 for all pixels with a change in water occurrence $\left(\Delta w_{m}\neq 0\right)$ within the total area of the SRD (i.e., D1 + D2 + D3) are shown in Fig. 9. Judging by the median of the pixels for each month (green line in Fig. 9), $\bar{w}$ has decreased in all months but October, agreeing with a general decrease in the runoff. October sees the most significant number of pixels increasing water occurrence when surface runoff is steadily increasing. However, the increase in water occurrence in October can be partly due to the starting of the leaf-off season that the water below vegetation appears in optical images.

**Fig. 10.**
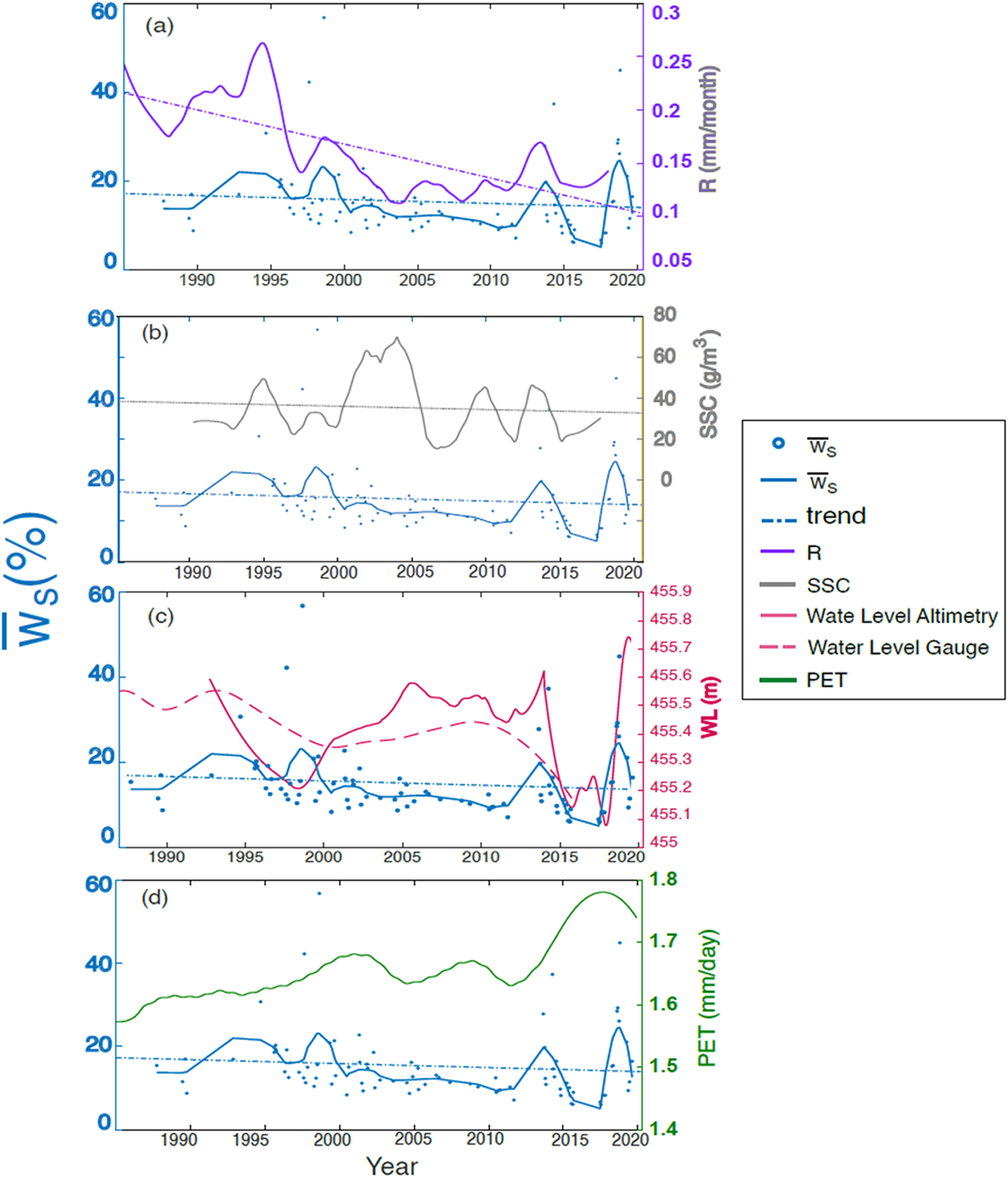
The relationship between ${\bar{w}}_{s}$ (left vertical axis) and (a) runoff, (b) suspended sediment concentration (SSC), (c) water level (altimetry and Babushkin gauging station), and (d) potential evapotranspiration (PET) in the mid-Delta. A Loess filter smoothes all data. The surface of reference for the gauge stations is the sea level and for the altimetric water levels is geoid GGMO2C (Tapley et al., 2005).

Regarding the main hydrological driver of changes in surface water occurrence $\left({\bar{w}}_{s}\right)$ in the SRD, we find a positive and significant (p < 0.05; Pearson) linear relationship between runoff and water occurrence in all three regions, and the highest coefficient of determination (*R*^2^_=_ 0.58) in the mid-region D2 (Fig. 10a, Fig. S6, and Table 2). The lower R^2^ in D1 is then since the river is not yet divided into distributaries. Therefore, the streams in D1 are permanent water bodies with high flow rates, and consequently, the water occurrence, irrespective of changes in river discharge, does not change much in this region. Specific extreme changes in water occurrence in the D2 region seem to replicate those in the runoff, such as 1995, 1998, and 2014. Furthermore, runoff shows a more pronounced long-term decreasing trend than water occurrence, regardless of the discharge station selected and different temporal moving windows of *R* (i.e., instantaneous and 5-day and 10-day averages of *R* before and after the images’ acquisition dates).

Regarding sediment, the long-term decrease in SSC has the same mildly decreasing trend as surface water occurrence (Fig. 10b, trend ≈ 0.0003). Nevertheless, the relationship of extremes in SSC to extremes in water occurrence is weak since bedload rather than the suspended load is generally responsible for planform migration as submerged bank areas are characterized by a considerable proportion of well-sorted bed-material sediment (i.e. sand) with a dominant fraction up to 125 μm (Pietroń et al., 2018). This is also supported by the data showing the termination of gravel and sands in Delta’s bed sediments downstream (Dong et al., 2019), whereas suspended sediment load is changed within 20–30% of the total inflow (Chalov et al., 2017b).

Furthermore, the lake water level does not influence surface water occurrence, even in region D3, where its effect should be most evident, signaling a low influence of the Lake’s backwater effect (Fig. 10c and Fig. S7). Finally, potential evapotranspiration (PET) appears to have a low influence compared to incoming runoff. Although PET has had an increasing trend during the last three decades (Fig. 10d), the short summer and low solar insolation in the area of the SRD weaken the effect of the PET on water occurrence compared to the effect of incoming runoff.

## 4. Discussion

We found a mild decrease in surface water occurrence in 51% of the SRD’s surface area between 1987 and 2020. However, water occurrence in the Delta varies spatially from 1987–2002 to 2003–2020, in the most affected areas decreasing by 20%, and in the most water-gaining areas increasing by 10%. The decrease happened across all regions of the delta D1, D2, and D3. This finding agrees with the general decrease in water occurrence reported for Northern Siberia during the same period (Borja et al., 2020), where permanent and seasonally flooded areas were also considered. Borja et al. (2020) attributed the decrease in surface water area to decreasing river discharge.

Furthermore, the changes in surface water occurrence are not heterogeneous throughout the Delta. For example, the percentage of pixels in the D2 region with decreasing water occurrence in the left (SW), right (NE), and central portions of the Delta are 44%, 51%, and 52%, respectively. These results are consistent with Chalov et al. (2017a) who show that maximum sediment aggregation before 1998 occurs in the NE and the central portion of the Delta, with some water bodies filled recently with sediment and contributing to the expansion of the Delta. Chalov et al. (2017a) also show higher relative suspended sediment retention in the central portion and the NE of the Delta, and although the sediment discharge is decreasing in the area, the surface of the Delta is rising by a velocity of 75 cm/year (Chalov et al., 2017a). In terms of the longitudinal change of the water occurrence, the percentage of the area showing a decrease in water occurrence in regions D1(River bifurcation), D2 (wetland-dominated area), and D3 (closer to the Lake) are 41%, 49%, and 45%, respectively indicating that water occurrence in wetland-dominated areas with low elevations is more influenced by river discharge.

Although our study shows a clear relationship between water occurrence and runoff, the relationship should be higher as the Selenga River is the only freshwater input into the Delta. More water discharge brings more sediment loads that lead to a gain in dry surfaces when accumulated. It is known that the Delta retains ~3000 tons/day of suspended sediments, an amount that outweighs the total flux of sediments to the entire Lake (Chalov et al., 2015). However, this may not be the case for the SRD under the period of study. Chalov et al. (2015) found that during 1983–2011, the correlation factor between the surface runoff and the suspended sediment concentration was only 0.16 (Chalov et al., 2015; Moragoda and Cohen, 2020). Furthermore, no significant overbank flow has been observed in the flood season after 2011 (Chalov et al., 2015), leading to no flooding from bank overflow, reducing an even stronger relationship between the runoff and water occurrence.

The weak relationship between water occurrence and water level in Lake Baikal can relate to the regulation of the lake by the Irkutsk dam on the main outflow of Lake Baikal – Angara River. Since the Irkutsk dam was created in 1959, lake water levels are more homogeneous than before (during the last three decades are within half a meter), and such changes may not imply considerable changes in water occurrence in the Delta.

Our results of water occurrence are also important to understand the biogeochemistry of the Delta. According to Chalov et al. (2017b), the Delta reduces heavy metals concentrations by 77–99% during moderate and high flow conditions. Furthermore, according to the observed 51% decrease of water occurrence, seasonally flooded regions have a crucial control on total metal concentrations’ change due to disconnectivity of smaller wetland-dominated channels (Shinkareva et al., 2019). In addition, wetlands act as active sinks for various metals depending on the ambient geochemical conditions such as the water pH, sediment mineralogy, organic matter, and oxygen availability (Pietroń et al., 2018). Finally, in agreement with Nienhuis et al. (2020), our results show that runoff may be the most important driver controlling water occurrence for coastal deltas with microtidal environments and experiencing minor variations of sea-level change. However, anthropogenic activities such as deforestation and damming may also affect runoff, so the real contribution to water occurrence changes in deltas is still subject to climatic and anthropogenic factors.

## 5. Conclusions

1. We find a mild decrease in surface water occurrence across 51% of the Selenga River Delta’s extent between 1987 and 2020. However, surface water occurrence varies spatially from 1987–2002 to 2003–2020; it decreases by 20% in the most affected areas and increases by 10% in the most water-gaining areas. The decreasing water occurrence occurs mostly in seasonally flooded regions and the increasing one on the outer bank of the Delta on the lake, probably land gain by sediment accumulation. The most considerable changes in water occurrence are found along the mainstream bends due to planform migration, resulting in a change in river course directions in the east of the Delta.
2. We have improved the standard methodology of determining the change in water occurrence by providing the training data for all available historical images in the Maximum Likelihood supervised classification. On a local scale, the training datasets in Deltas with complex structures lead to an accurate map of surface water occurrence and its change.
3. There is a significant relationship between surface water occurrence and surface runoff (R^2^_=_ 0.56). The best fit between these two parameters was observed for the Delta’s inner zone due to the synchronized peaks in extreme precipitation and runoff in 1987–2020. Although the long-term trend of surface water occurrence is similar to that of suspended sediment concentration, the peaks are not synchronized. Furthermore, surface water occurrence does not correlate with the Lake water level and potential evapotranspiration.

The change in water occurrence between 1987–2002 and 2003–2020 shows channel platforms and stream network change. The bend migration and avulsion identified here illustrate in-channel processes happening at the local scale (channel bends) due to the evolution of the Delta structure (partitioning of water between sectors). However, the SRD has shown a relatively stable long-term surface water occurrence under decreasing precipitation and runoff and increasing temperature during the last four decades. We expect that any modification of the river flow through upstream damming and water diversion compounded by climate change will impact water occurrence in the future.

## Supplementary Material

Supplement1

## Figures and Tables

**Table 1:** The confusion matrix of this study and Pekel et al. (2016) compared to the study by Berhane et al. (2018) showing the number of pixels per class. All images are from June 2011. The overall accuracy is written in the intersection cell of the User’s Accuracy (UA) and Producer’s accuracy (PA) metrics.

1		Berhane et al. (2018)				
2	This study		Water (1)	Non-water (0)	Total	UA
3		Water (1)	3666	595	4261	***0.86***
4		Non-water (0)	1334	4405	5739	0.77
5		Total	5000	5000	10,000	
6		PA	***0.73***	0.88		***0.81***
7	Pekel et al. (2016)	Water (1)	2688	382	3070	***0.88***
8		Non-water (0)	2306	4617	6923	0.67
9		Total	4994	4999	9993	
10		PA	***0.54***	0.92	0	***0.73***

**Table 2:** Statistics of the linear regressions between ${\bar{w}}_{s}$ and runoff (***R***), SSC, water level (***WL***) in Lake Baikal, and potential evapotranspiration (PET) in D1, D2, and D3. Bold values are the highest of all regions. We selected ***R*** data on the images’ acquisition days, SSC with a 2-week delay, the Lake water level data nearest to the image acquisitions date, and PET in the same month.

	${\bar{w}}_{s}$ vs. *R*		${\bar{w}}_{s}$ vs. *SSC*		${\bar{w}}_{s}$ vs. *WL* (Altimetry)		${\bar{w}}_{s}$ vs. *PET*	
	R^2^	p-value	R^2^	p-value	R^2^	p-value	R^2^	p-value

D1 (River bifurcation)	0.20	<< 0.001	0.02	0	0	0.64	0.04	0.08
D2 (Mid-Delta)	***0.58***	**<< 0.001**	0.01	0.70	0.06	0.03	0.01	0.28
D3 (Lake-ward)	0.09	0.01	0	0.35	0	0.86	0.20	0
Entire Delta	0.02	0.20	0	0.60	0	0.63	0.19	0

## Data Availability

The Landsat Level-2 Surface Reflectance data were obtained from the U.S. Geological Survey (https://earthexplorer.usgs.gov/) and are freely available On-demand. They are described in these citation references: Masek et al. (2006), Vermote et al. (2016), p. 8. Digital Object Identifier (DOI) for L8, L7 and L4 data respectively: [https://doi.org/10.5066/F78S4MZJ], [https://doi.org/10.5066/F7Q52MNK], [https://doi.org/10.5066/F7KD1VZ9]. The temperature and precipitation data for the region of the Delta were obtained from the gridded data sets of the CRU of the Climatic Research Unit and are available in these in-text data citation references: Hulme (1992) [with Open Database License: http://opendatacommons.org/licenses/odbl/1.0/ and Database Contents License: http://opendatacommons.org/licenses/dbcl/1.0/ under conditions of Attribution and Share-Alike: http://opendatacommons.org/licenses/odbl/summary/], Hulme et al. (1998). The dynamic land cover map of the Copernicus Global Land Service at 100-m resolution (CGLS-LC100) is available at this in-text citation reference: Buchhorn et al. (2019). For Lake Baikal water level, we used two gauge stations of the International Data Centre on Hydrology of Lakes and Reservoirs (Hydrolare) from 1963 to 2015, Babushkin station and Ushkanij station, together with the processed satellite altimetric data of the Hydroweb service (Crétaux et al., 2011) available since 1992 and continuously updated. The daily discharge data (Mostovoi and Kabansk stations) was obtained from the Russian Federal Service for Hydrometeorology and Environmental Monitoring (Roshydromet) and is not accessible to the public or the research community.
