# Supplementary material for: Drivers and extent of surface water occurrence in the Selenga River Delta, Russia
Source: J Hydrol Reg Stud. Author manuscript; Available in PMC 2022 Dec 1. (PMC9067400; doi:10.1016/j.ejrh.2021.100945)
Supplement: Supplement1 [file NIHMS1751663-supplement-Supplement1.zip › 1-s2.0-S2214581821001749-mmc1.docx]

Supplementary

Drivers and Extent of Surface Water Occurrence in the Selenga River Delta, Russia

Saeid Aminjafari^a^, Ian Brown^a^, Sergey Chalov^b^, Marc Simard^c^, Charles R. Lane^d^, Jerker Jarsjö^a^, Mehdi Darvishi^a^, Fernando Jaramillo^a,e^

^a^Department of Physical Geography and Bolin Centre for Climate Research, Stockholm University, Stockholm, SE–106 91, Sweden. saeed.aminjafari@natgeo.su.se, Ian.Brown@natgeo.su.se, jerker.jarsjo@natgeo.su.se, mehdi.darvishi@natgeo.su.se

^b^Faculty of Geography, Lomonosov Moscow State University, Moscow, 119991, Russia. srchalov@geogr.msu.ru

^c^Jet Propulsion Laboratory, California Institute of Technology, Pasadena, CA, 91109, USA. marc.simard@jpl.nasa.gov

^d^Office of Research and Development, U.S. Environmental Protection Agency, Athens, Georgia, 45268, USA. Lane.Charles@epa.gov

^e^Baltic Sea Centre and Stockholm Resilience Center, Stockholm University, Stockholm, SE-106 91, Sweden. fernando.jaramillo@natgeo.su.se

corresponding author: Saeid Aminjafari (saeed.aminjafari@natgeo.su.se)


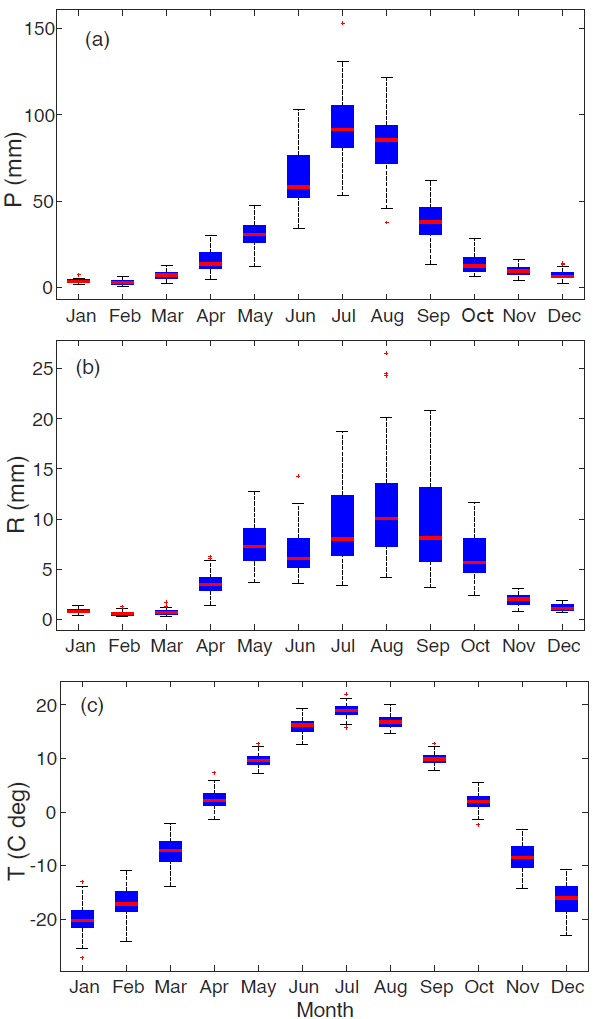


**Figure S1.** Distributions of **a)** total monthly precipitation (P; mm/month), **b)** runoff (R; mm/month), and **c)** average monthly temperature (T, oC) over the period 1985-2019. For the runoff calculations, we divided discharge data at station Mostovoi by the upstream hydrological basin of 440,200 km2, of which 67% falls in Mongolia and 33% in Russia. Red crosses represent the outliers.


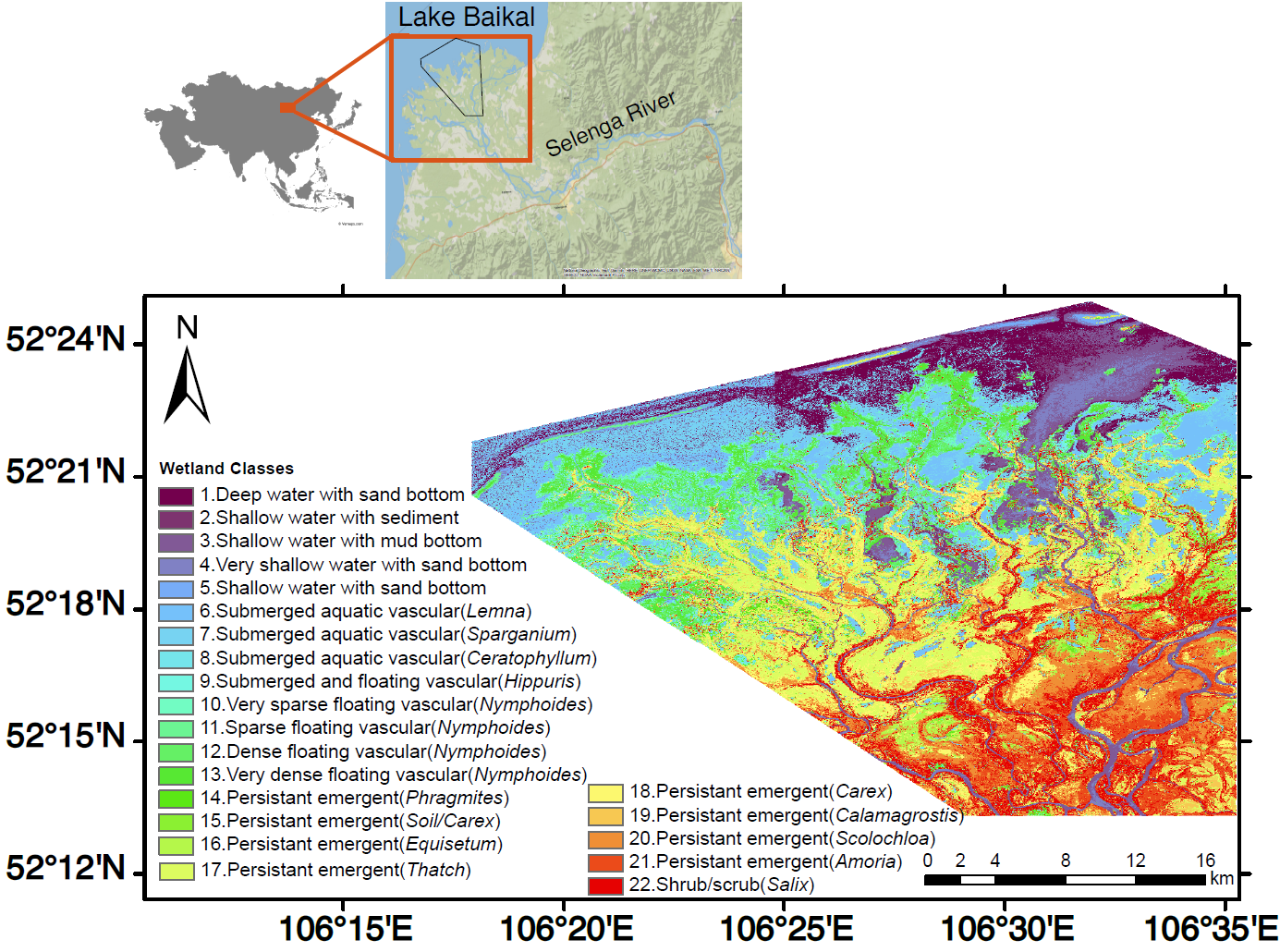


Figure S2. High-resolution multispectral classification of the Selenga River Delta (Berhane et al. 2018), (25 Jun 2011 and 3 Jul 2011).


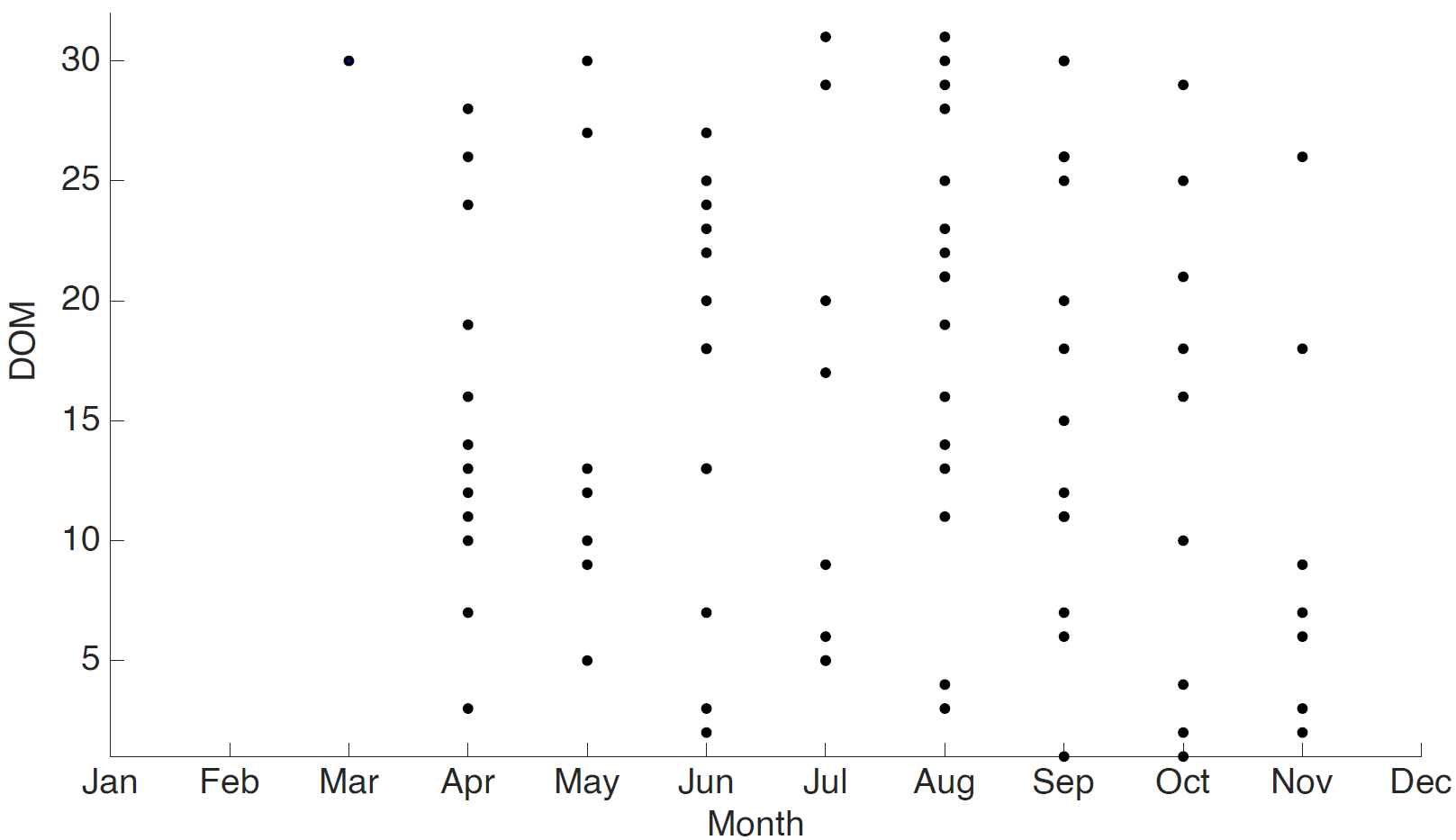


Figure S3. The number of Landsat images used in this study per month. Y-axis shows the day of the month (DOM)


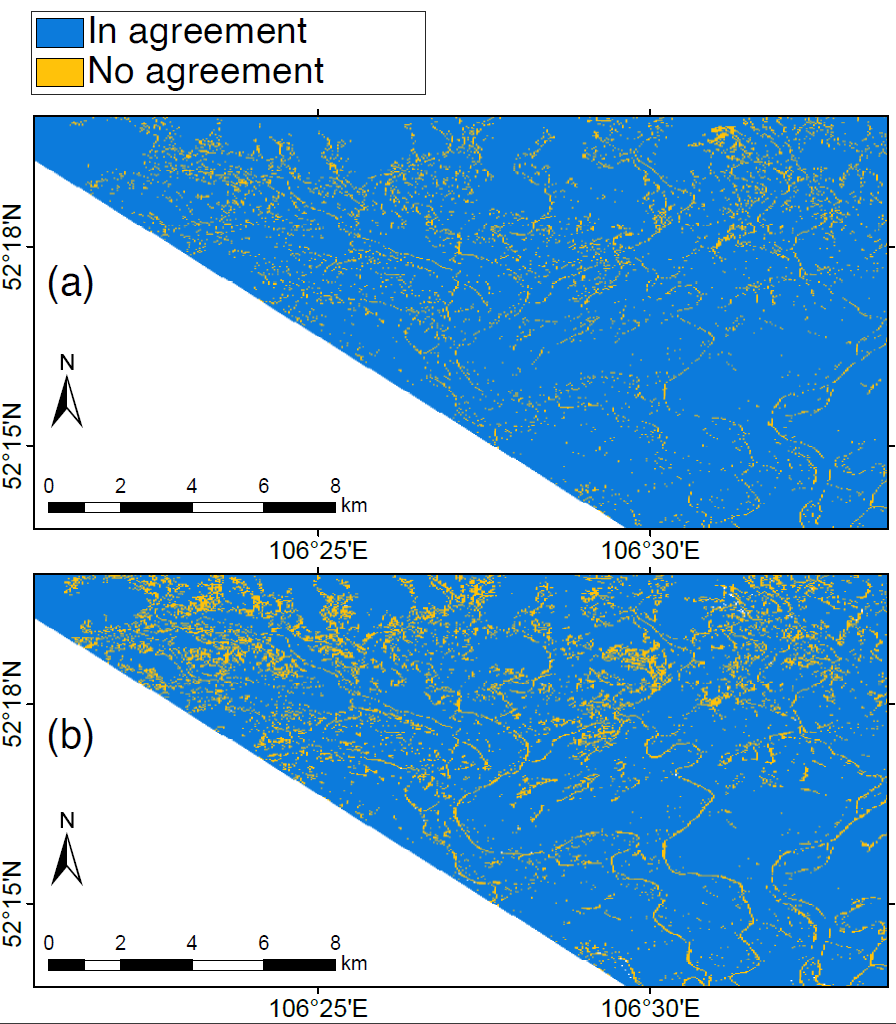


**Figure S4.** Agreement of binary classification by Pekel et al. (2016) and our study, with the reference water body delineation by Berhane et al. (2018) in the Delta in June 2011 that was degraded from 2-m to 30-m resolution by the nearest neighbor method; **(a)** agreement maps of our study **(b)** and Pekel et al. (2016).


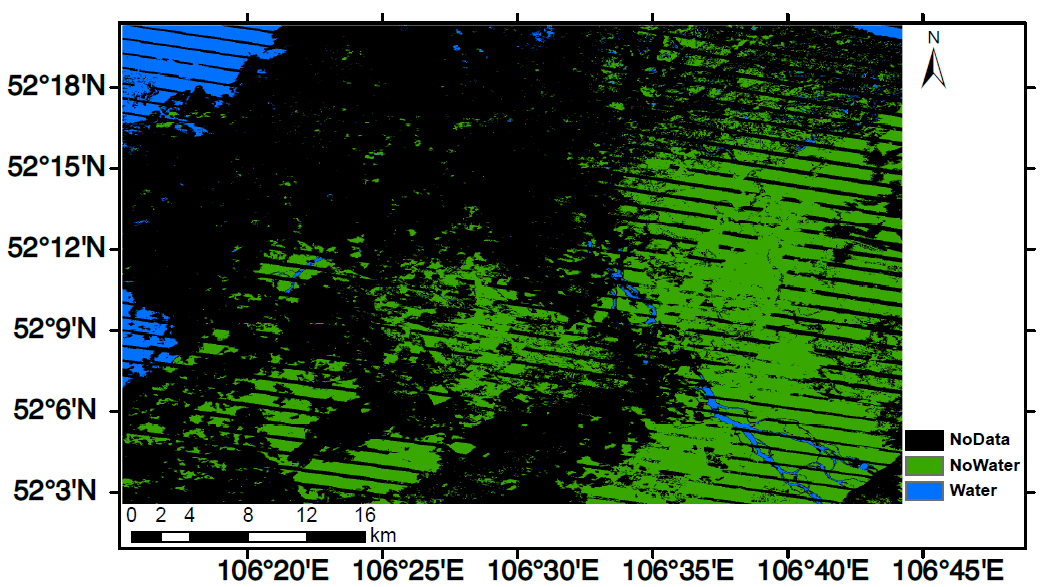


Figure S5. One example of the contaminated class image of the global study by Pekel et al. (2016). High cloud coverage and Scan Line Corrector (SLC) errors of Landsat-7 led to pixels with a no-data value that can contribute to spatially biased water occurrence in the final averaged map.


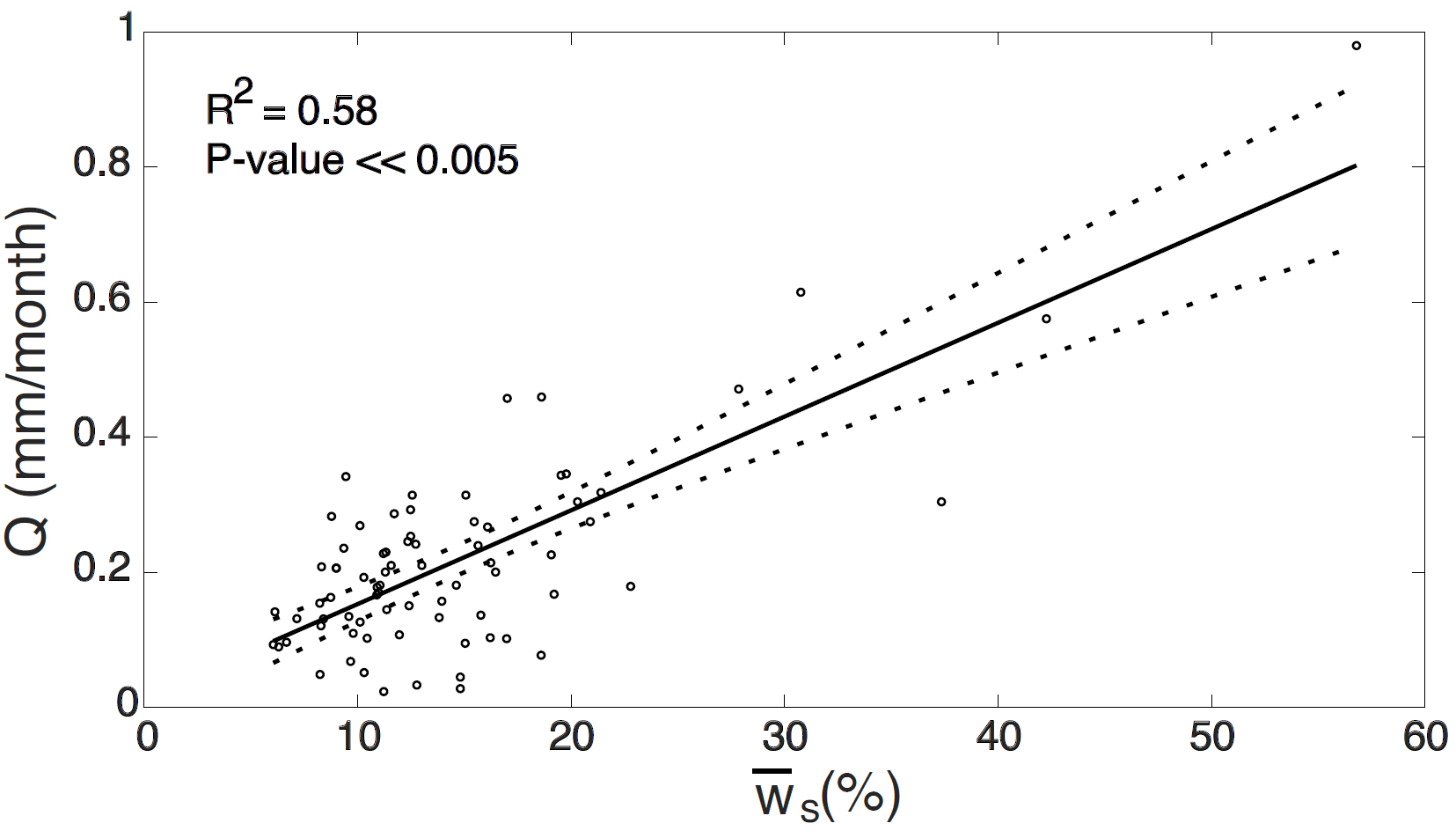


Figure S6. Scatter plot of the surface runoff (Q) vs the time series of the surface water occurrence ${\bar{\mathbf{w}}}_{\mathbf{s}}$. There is a positive and significant (p < 0.05) linear regression between Q and ${\bar{\mathbf{w}}}_{\mathbf{s}}$ that is significant throughout the Delta and highest (R^2^=0.58) in the central portion (D2).


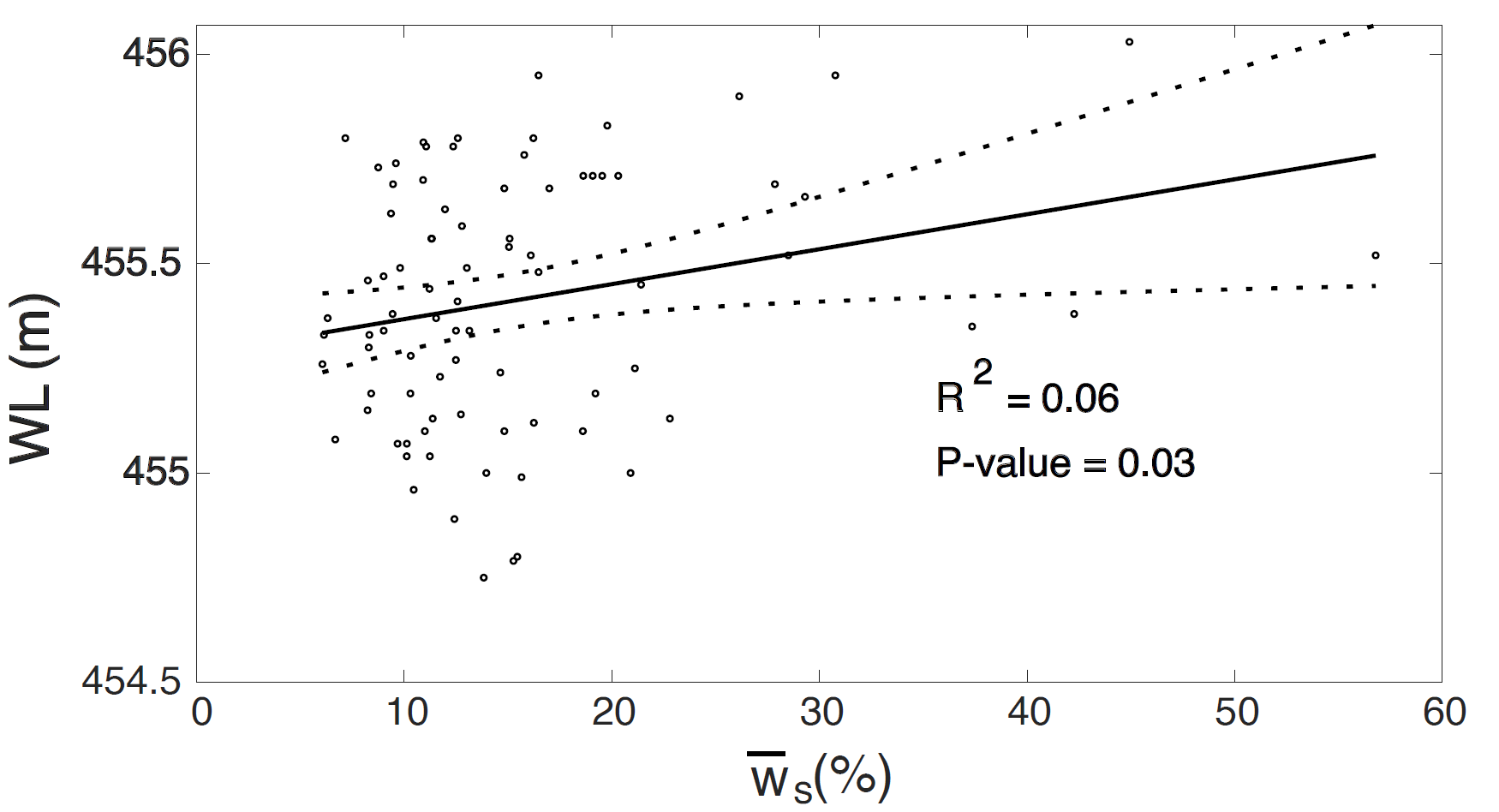


Figure S7. Scatter plot of the Lake Water Level (WL) vs the time series of the surface water occurrence ${\bar{\mathbf{w}}}_{\mathbf{s}}$. There is not a significant (p < 0.05) linear regression between WL and ${\bar{\mathbf{w}}}_{\mathbf{s}}$ in the central portion of the Delta (D2).

Table S1. Classification accuracy for Landsat images 1987/09/25, 1995/09/15, 2005/09/26, and 2018/09/30

| 1987-09-25 | Overall accuracy (%) | 99.57 |
| --- | --- | --- |
|  | Kappa Coefficient | 0.98 |
| 1995-09-15 | Overall accuracy (%) | 98.99 |
|  | Kappa Coefficient | 0.95 |
| 2005-09-26 | Overall accuracy (%) | 99.82 |
|  | Kappa Coefficient | 0.99 |
| 2018-09-30 | Overall accuracy (%) | 98.88 |
|  | Kappa Coefficient | 0.91 |

Table S2. Definition and mathematical formulation of the accuracy assessment parameters

| Accuracy Assessment Parameter | Definition |
| --- | --- |
| User's Accuracy (UA) | The number of pixels correctly classified as water (TP: True Positive) is divided by the whole number of pixels classified as water. This parameter is calculated based on the pixels that have been classified as water, but according to the reference image (here the reference image is Berhane et al. (2018)), they belong to the non-water class (false positive pixels, FP). |
| Producer's Accuracy (PA) | The number of pixels correctly classified as water (TP: True Positive) is divided by the number of all water classes in the reference image. This parameter is calculated based on the pixels that have been classified as non-water, but according to the reference image, they belong to the water class (false negative FN). |
| Overall Accuracy (OA) | The division of all the correctly classified pixels by the whole number of pixels. |
| F-score | The harmonic mean of user's accuracy and producer's accuracy:  $\boldsymbol{F}\mathbf{-}\boldsymbol{score}\mathbf{=2*}\frac{\boldsymbol{UA}\mathbf{*}\boldsymbol{PA}}{\boldsymbol{UA}\mathbf{+}\boldsymbol{PA}}$  This parameter does not consider the true negative (TN, pixels correctly detected as non-water) and is not suitable for unbalanced classes and binary classification (Chicco & Jurman, 2020). |
| Kappa | Similar to the overall accuracy but considering agreements between the two images, where $\boldsymbol{p}$ is the probability that two class images agree on water/non-water classes (Foody, 2020):  $\boldsymbol{Kappa=}\frac{\boldsymbol{OA-p}}{\boldsymbol{1-p}}$ |
| Matthews Correlation Coefficient (MCC) | this parameter is calculated by this equation:  $\boldsymbol{MCC=}\frac{\boldsymbol{TP*TN-FP*FN}}{\sqrt{\left( \boldsymbol{TP+FP} \right)\boldsymbol{*}\left( \boldsymbol{TP+FN} \right)\boldsymbol{*}\left( \boldsymbol{TN+FP} \right)\boldsymbol{*(TN+FN)}}}$  The MCC is widely used for comparing binary classification, and since we consider all the combinations of True, False, Positive, and Negative pixels in the formula, it works well with unbalanced classes (Boughorbel et al., 2017). This parameter varies between -1 (total disagreement) and +1 (perfect agreement). |

Table S3. The number of images used in the study per year. The numbers in each cell show the day of the month.

|  | Jan | Feb | Mar | Apr | May | Jun | Jul | Aug | Sep | Oct | Nov | Dec |
| --- | --- | --- | --- | --- | --- | --- | --- | --- | --- | --- | --- | --- |
| 1987 |  |  |  |  |  |  |  |  | 25 |  |  |  |
| 1988 |  |  |  |  |  |  |  |  |  |  |  |  |
| 1989 |  |  |  |  |  | 18 |  | 21 | 30 |  |  |  |
| 1990 |  |  |  |  |  |  |  |  |  |  |  |  |
| 1991 |  |  |  |  |  |  |  |  |  |  |  |  |
| 1992 |  |  |  |  |  |  |  |  |  |  | 09 |  |
| 1993 |  |  |  |  |  |  |  |  |  |  |  |  |
| 1994 |  |  |  |  |  |  |  |  | 12 |  |  |  |
| 1995 |  |  |  |  |  |  |  | 14,30 | 15 | 01 |  |  |
| 1996 |  |  |  | 26 |  | 13 | 31 |  | 01 |  |  |  |
| 1997 |  |  |  | 13 |  |  |  | 03,19 | 20 |  | 07 |  |
| 1998 |  |  |  | 16 |  | 03 | 05 | 22 |  |  |  |  |
| 1999 |  |  |  |  | 05 | 22 |  | 25 | 26 |  |  |  |
| 2000 |  |  |  |  |  | 24 |  | 11 |  |  |  |  |
| 2001 |  |  |  | 24 | 10 | 27 | 29 |  |  |  | 02,18 |  |
| 2002 |  |  |  | 11 | 13 |  |  |  | 18 |  |  |  |
| 2003 |  |  |  |  |  |  |  | 04 |  |  |  |  |
| 2004 |  |  |  |  |  |  | 05 |  | 07 | 25 | 26 |  |
| 2005 |  |  |  | 03,19 |  |  |  |  | 26 |  |  |  |
| 2006 |  |  |  |  |  | 25 |  | 28 |  |  |  |  |
| 2007 |  |  |  |  | 27 |  |  |  |  |  |  |  |
| 2008 |  |  |  |  |  |  |  |  |  | 04 |  |  |
| 2009 |  |  |  | 14 |  |  |  |  |  |  |  |  |
| 2010 |  |  |  |  |  | 20 | 06 | 23 |  | 10 |  |  |
| 2011 |  |  |  |  |  | 07 |  |  | 11 |  |  |  |
| 2012 |  |  |  |  |  |  |  |  |  |  |  |  |
| 2013 |  |  |  |  |  |  |  | 31 |  | 02,18 | 03 |  |
| 2014 |  |  |  | 12,28 | 30 |  | 17 |  |  | 21 | 06 |  |
| 2015 |  |  | 30 |  |  | 02,18 | 20 | 21 | 06 |  |  |  |
| 2016 |  |  |  |  |  |  |  |  |  |  |  |  |
| 2017 |  |  |  |  |  | 23 | 09 |  | 11 | 29 |  |  |
| 2018 |  |  |  | 07 | 09 |  |  | 13,29 | 30 | 16 |  |  |
| 2019 |  |  |  | 10 | 12 | 13 |  | 16 |  |  |  |  |

Table S4. The Number of Pixels per landcover and the number of pixels with no change in surface water occurrence in the corresponding land cover ($\bar{\boldsymbol{\Delta w}}\boldsymbol{\neq0}$).

| Land Cover | Number of Pixels with $\bar{\boldsymbol{\Delta w}}\boldsymbol{\neq0}$ | Number of Pixels per Land Cover |
| --- | --- | --- |
| Closed Forest Deciduous Needle Leaf (*CFDNL*) | 8201 | 9386 |
| Closed Forest Not matched with any classes (*CFN*) | 48533 | 52925 |
| Herbaceous vegetation (*Herb*) | 261683 | 334843 |
| Open Forest Deciduous Needle Leaf (*OFDNL*) | 951 | 1143 |
| Open Forest Not matched with any classes (*OFN*) | 138628 | 147202 |
| Bare Land and Sediments (*Sand Bar*) | 2007 | 2046 |
| Shrubs | 3894 | 3929 |
| Wetland herbaceous | 73872 | 78084 |
| Permanent Water | 107903 | 592437 |
| Seasonal Water | 250194 | 252062 |
| Land | 338522 | 522034 |
| Total | 696621 | 1366533 |

**References**

Boughorbel, S., Jarray, F., El-Anbari, M., 2017. Optimal classifier for imbalanced data using Matthews Correlation Coefficient metric. PLoS One 12 (6), e0177678. doi:10.1371/journal.pone.0177678.

Chicco, D., Jurman, G., 2020. The advantages of the Matthews correlation coefficient (MCC) over F1 score and accuracy in binary classification evaluation. BMC Genom. 21 (1), 1–13. doi:10.1186/%2Fs12864-019-6413-7.

Foody, G.M., 2020. Explaining the unsuitability of the kappa coefficient in the assessment and comparison of the accuracy of thematic maps obtained by image classification Remote Sens Environ. 239, 111630. doi:10.1016/j.rse.2019.111630.
