# Supplementary figures and images for: Drivers and extent of surface water occurrence in the Selenga River Delta, Russia
Source: J Hydrol Reg Stud. Author manuscript; Available in PMC 2022 Dec 1. (PMC9067400; doi:10.1016/j.ejrh.2021.100945)

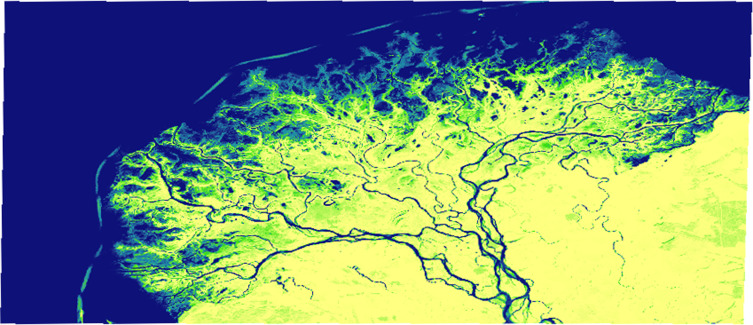

Supplement: Supplement1 [file NIHMS1751663-supplement-Supplement1.zip › 1-s2.0-S2214581821001749-mmc3.jpg]

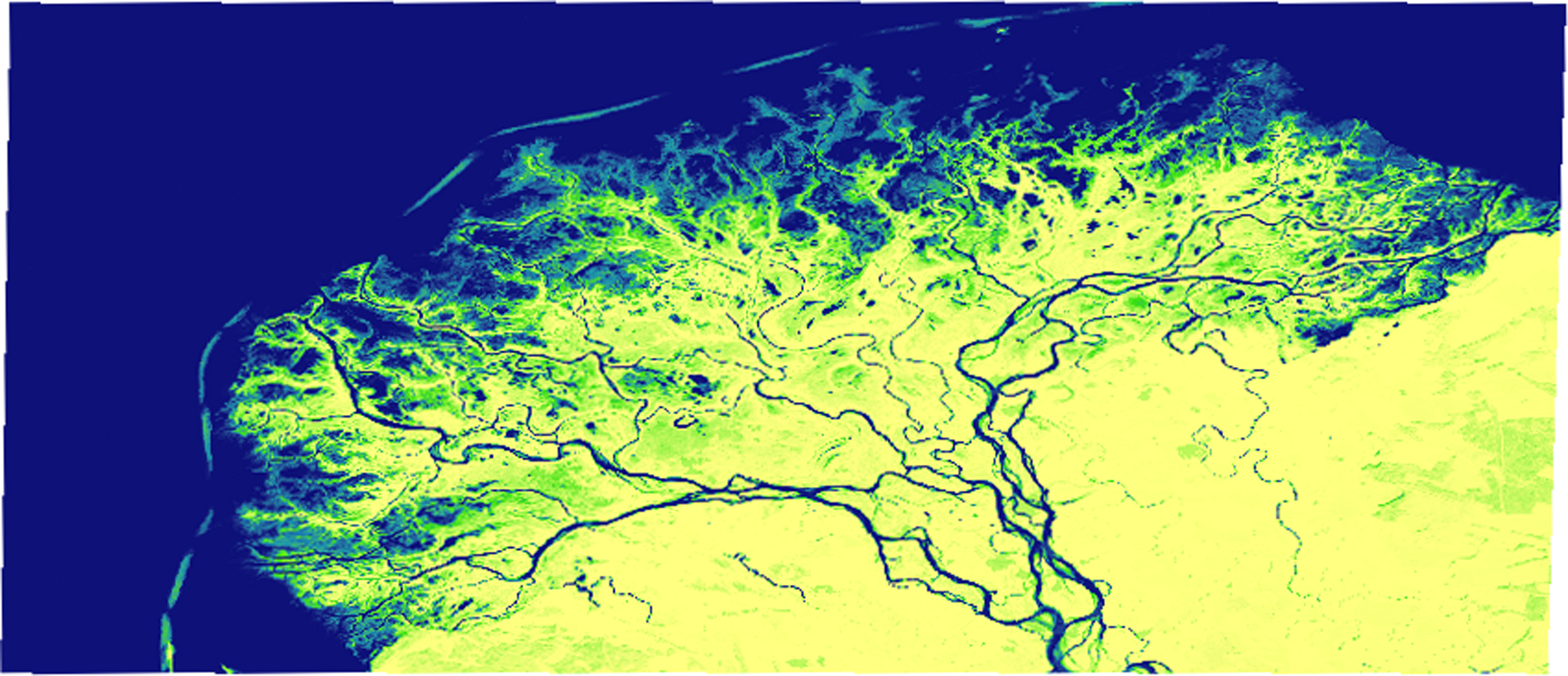

Supplement: Supplement1 [file NIHMS1751663-supplement-Supplement1.zip › 1-s2.0-S2214581821001749-mmc3_lrg.jpg]
